# Hypoxic-Ischemic Insult Alters Polyamine and Neurotransmitter Abundance in the Specific Neonatal Rat Brain Subregions

**DOI:** 10.1021/acschemneuro.4c00190

**Published:** 2024-07-26

**Authors:** Hynek Mácha, Dominika Luptáková, Ivo Juránek, Per E. Andrén, Vladimír Havlíček

**Affiliations:** † Institute of Microbiology of the Czech Academy of Sciences, Vídeňská 1083, Prague 142 00, Czech Republic; ‡ Department of Analytical Chemistry, Faculty of Science, Palacký University, 17. listopadu 12, Olomouc 771 46, Czech Republic; § Department of Pharmaceutical Biosciences, Spatial Mass Spectrometry, Science for Life Laboratory, Uppsala University, Husargatan 3, Uppsala 75124, Sweden; ∥ Biomedical Research Center, Slovak Academy of Sciences, Dubravska Cesta 9, 845 05 Bratislava, Slovak Republic; ⊥ Centre of Experimental Medicine, Slovak Academy of Sciences, Dúbravská Cesta 9, 841 04 Bratislava, Slovak Republic

**Keywords:** neonatal hypoxic-ischemic encephalopathy, MALDI mass spectrometry imaging, metabolome dynamics, histology, cerebrospinal fluid, polyamines, neurotransmitters

## Abstract

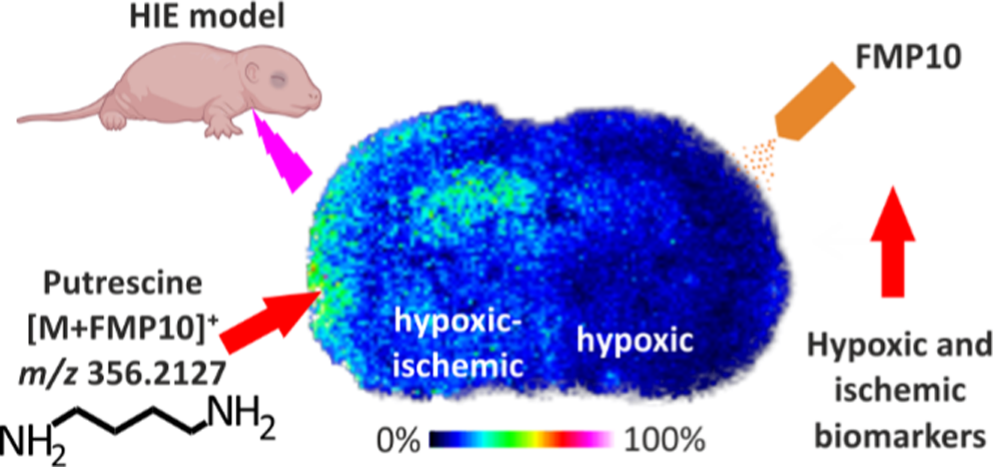

Neonatal hypoxic-ischemic (HI) brain insult is a major cause of neonatal mortality and morbidity. To assess the underlying pathological mechanisms, we mapped the spatiotemporal changes in polyamine, amino acid, and neurotransmitter levels, following HI insult (by the Rice–Vannucci method) in the brains of seven-day-old rat pups. Matrix-assisted laser desorption/ionization mass spectrometry imaging of chemically modified small-molecule metabolites by 4-(anthracen-9-yl)-2-fluoro-1-methylpyridin-1-ium iodide revealed critical HI-related metabolomic changes of 22 metabolites in 14 rat brain subregions, much earlier than light microscopy detected signs of neuronal damage. For the first time, we demonstrated excessive polyamine oxidation and accumulation of 3-aminopropanal in HI neonatal brains, which was later accompanied by neuronal apoptosis enhanced by increases in glycine and norepinephrine in critically affected brain regions. Specifically, putrescine, cadaverine, and 3-aminopropanal increased significantly as early as 12 h postinsult, mainly in motor and somatosensory cortex, hippocampus, and midbrain, followed by an increase in norepinephrine 24 h postinsult, which was predominant in the caudate putamen, the region most vulnerable to HI. The decrease of γ-aminobutyric acid (GABA) and the continuous dysregulation of the GABAergic system together with low taurine levels up to 36 h sustained progressive neurodegenerative cellular processes. The molecular alterations presented here at the subregional rat brain level provided unprecedented insight into early metabolomic changes in HI-insulted neonatal brains, which may further aid in the identification of novel therapeutic targets for the treatment of neonatal HI encephalopathy.

## Introduction

Neonatal hypoxic-ischemic (HI) insult of the brain is a major cause of newborn mortality and morbidity,^1^ with an incidence of approximately 2–3 episodes per 1000 live births in developed countries.^2^ Cerebral HI insult is primarily caused by the brain lacking sufficient blood, oxygen, and glucose supplies.^3^ The pathology typically evolves in three phases: (I) primary energy metabolism failure due to the lack of oxygen; (II) apparent recovery of energy metabolism after brain reoxygenation; and (III) secondary energy metabolism failure under normoxia due to failing mitochondria,^4^ followed by cellular neurodegeneration^5^ in susceptible brain regions.^6,7^ Changes in metabolic pathways in the affected brain regions can reflect the early stages of an injurious process,^8^ and specific metabolites appearing in cerebrospinal fluid (CSF) may be indicative.^9^

To investigate the mechanisms of neonatal brain HI injury, a limited number of various experimental models has been developed.^10,11^ One, applied in the study, is the Rice–Vannucci model, which involves permanent unilateral ligation of the common carotid artery of 7-day-old mice or rats and the animals’ subsequent exposure to systemic hypoxia.^12^ The pathophysiology of neonatal brain HI injury has been studied by magnetic resonance imaging (MRI)^13^ and magnetic resonance spectroscopy (MRS),^14^ which are inherently limited in their dynamic analytical range and restricted to paramagnetic nuclei. These two *in vivo* techniques allowed for qualitative and quantitative assessment of abundant metabolite classes, *i.e.*, lipids and amino and carboxylic acids.^15^ Of note, MRI and MRS have also demonstrated the extent of changes in lactate- and phosphate-containing metabolites and the development of cytotoxic and vasogenic edema,^16^ as well as a decrease in the levels of taurine, glutathione, total creatine, and myo-inositol^17^ in HI-affected regions. Specific imaging information on low abundance proteins can be provided by immunochemical labeling,^18^ as demonstrated on the acute periventricular white matter and oligodendroglial injuries due to the loss of basic myelin protein.^19^

On the contrary, label-free mass spectrometry imaging (MSI) enables in one experiment the imaging and quantitation of thousands of molecules providing detailed spatiotemporal analysis in the tissues.^20^ Despite several matrix-assisted laser desorption ionization (MALDI) MSI studies on HI neonatal brain injury,^17,21,22^ understanding alterations on the level of neurotransmitter and polyamine pathways and amino acids has been limited. Lipids, such as *N*-acetylphosphatidylethanolamine, and the gangliosides, GM2 and GM3, were localized in the HI-affected regions, including motor and somatosensory cortex (MCtx and SCtx) and caudate putamen (CPu), along with decreased adenosine mono- and diphosphate (AMP and ADP) levels.^16^ Glutamate and acetylcholine showed decreased levels specifically in the hippocampus (Hipp) and amygdala (Amy) under therapeutic hypothermia.^23^ Many molecular targets have poor ionization efficiency in the classical MALDI setup and remain hidden in the background chemical noise. In efforts to assist in the identification of new targets for treating HI injuries, we used chemical derivatization of key metabolites by 4-(anthracen-9-yl)-2-fluoro-1-methylpyridin-1-ium iodide (FMP-10), followed by MALDI-MSI.^20^ In this report, we discuss the region-specific metabolic alterations observed during various phases of the evolving injury that are critically involved in HI neonatal brain pathology.

## Results

### Three Temporal Phases of Metabolic Alterations were Triggered by HI Insult

**Figure 1:**
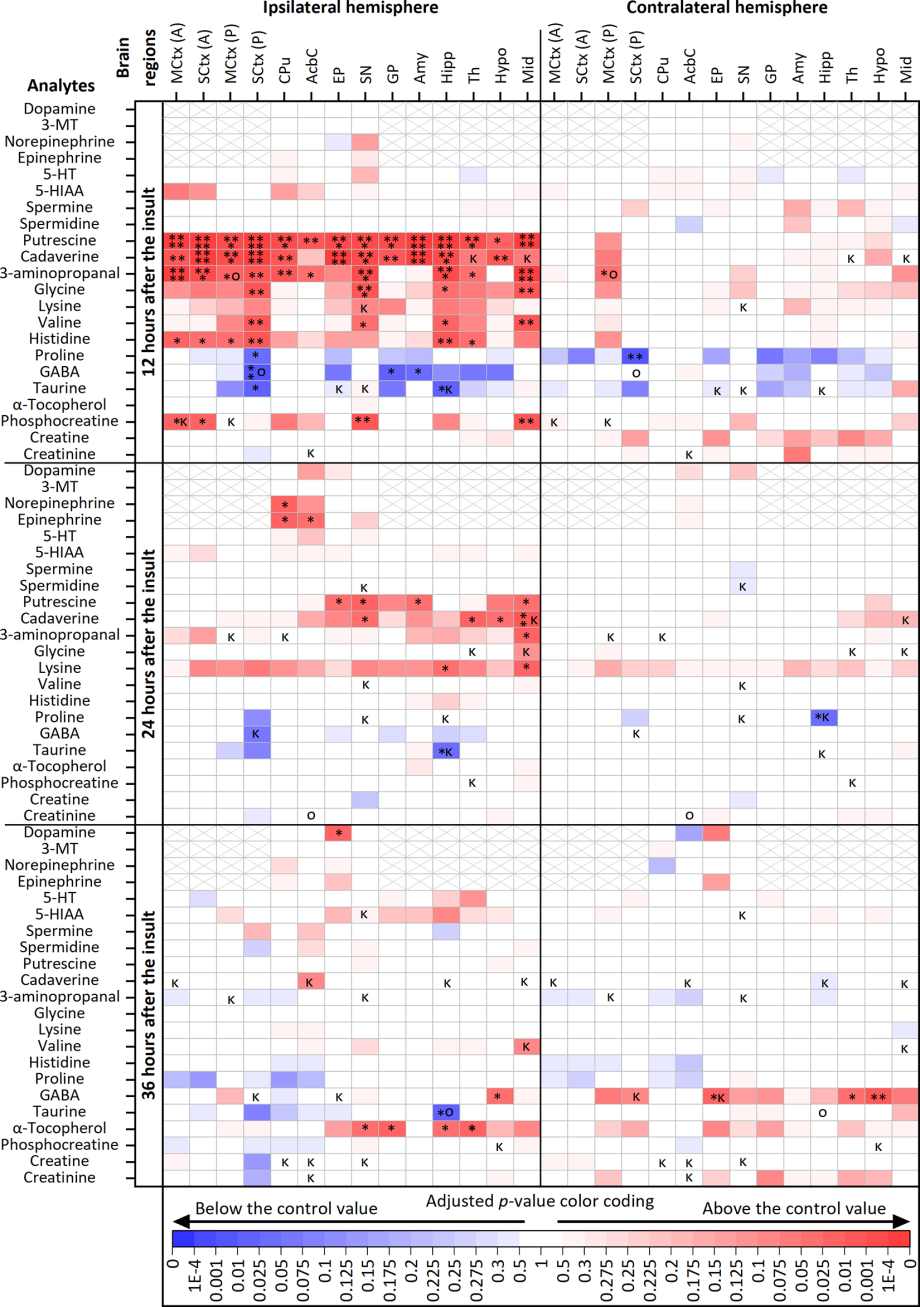
Metabolomic changes in the neonatal rat brain under HI insult. Heatmaps showing changes in 22 detected metabolites 12, 24, and 36 h after the onset of the insult (upper, middle, and lower panels, respectively). Increases and decreases in the levels of each metabolite in the indicated regions of the ipsilateral and contralateral hemispheres of HI-insulted animals are shown (in the left and right panels, respectively) relative to those in sham animals by red and blue rectangles, respectively (*n* = 4–6 biological replicates in each group; for details, see the Statistics section in the Materials and Methods section). The color intensity provides semiquantitative indications of *p* values of differences, calculated either by two-way ANOVA (no marks in boxes) or one-way ANOVA (ο) with Tukey’s multiple comparisons post hoc test or by Kruskal–Wallis (κ) with Dunn’s multiple comparison test, depending on whether the data met normality of distribution criteria: *p ≤ 0.05; **p ≤ 0.01; ***p ≤ 0.001; ****p ≤ 0.0001. Abbreviations: MCtx (A), anterior motor cortex; SCtx (A), anterior somatosensory cortex; MCtx (P), posterior motor cortex; SCtx (P), posterior somatosensory cortex; CPu, caudate putamen; AcbC, accumbens nucleus core; EP, entopeduncular nucleus; SN, substantia nigra; GP, globus pallidus; Amy, amygdala; Hipp, hippocampus; Th, thalamic region; Hypo, hypothalamic region; Mid, midbrain; 3-MT, 3-methoxytyramine; 5-HT, 5-hydroxytryptamine; 5-HIAA, 5-hydroxyindoleacetic acid; GABA, γ-aminobutyric acid.

The molecular mechanisms underlying neuronal damage due to HI insult were recorded by a bimodal imaging workflow (Figure S1), which enabled the visualization of time-dependent and region-specific metabolomic alterations and cellular changes in the neonatal rat brain (Figure 1). Fluctuations in 22 compounds, including several amino acids, polyamines, and neurotransmitters, were detected in 14 brain regions, including cortical subregions, basal ganglia, Hipp, thalamic region (Th), and midbrain (Mid), using MALDI-MSI analyses of brain sections collected 12, 24, and 36 h after the onset of the HI insult (hereafter, post-HII). Three types of temporally resolved and HI-evoked brain metabolomic alterations include (i) changes reflecting the acute metabolic response to the HI insult with prompt neonatal brain recovery, (ii) subacute metabolomic changes, and (iii) delayed metabolic changes.

**Figure 2:**
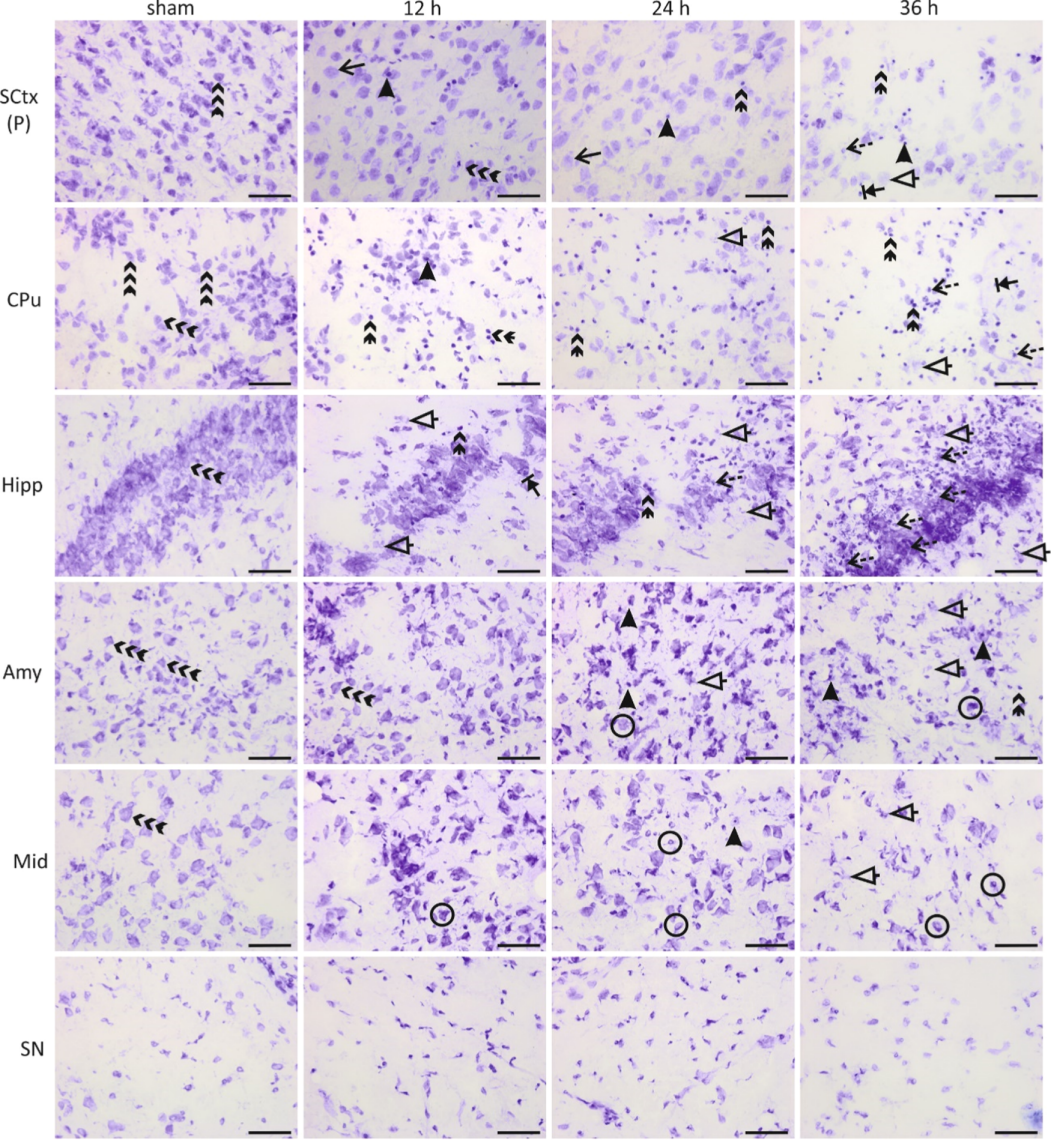
Histological images of the neonatal rat brain ipsilateral hemisphere after HI insult. Nissl-stained sections of the ipsilateral hemisphere, consecutive to those used for MALDI-MSI analysis, illustrating changes relative to those of sham animals (far left panels) 12, 24, and 36 h after insult, in the following regions: posterior somatosensory cortex [SCtx (P)], caudate putamen (CPu), hippocampus (Hipp), amygdala (Amy), midbrain (Mid), and substantia nigra (SN). The scale bar is 50 μm. Triple arrows, neurons; double arrows, apoptotic cells; circles, shapeless/shrunk cells; dotted arrows, necrotic cells; arrows, cell swelling; arrowheads, pyknotic nucleus; stop arrows, continuum cells; and triangle arrows, neuronal debris.

Histological examination of the Nissl-stained neonatal rat brain sections revealed no substantial cellular damage 12 h post-HII, as determined by light microscopy (Figure 2). On the contrary, molecular alterations preceded signs of tissue deterioration, which evolved gradually starting 12 h post-HII with symptoms of neurodegeneration in the SCtx, CPu, Hipp, and Amy, including apoptotic bodies and pyknotic nuclei in the CA1 region of the Hipp and the dorsal CPu of the ipsilateral (IL) hemisphere. The symptoms included cell swelling and shapeless cells with further deterioration by 24 h post-HII grading to apoptosis and necrosis. Later, at 36 h, a mixed type of neuronal death was observed, forming a continuum of states, accompanied by massive neuronal loss. Cell degeneration reduced cell density and led to the accumulation of neuronal debris within the affected regions. Signs of neurodegeneration were not displayed in substantia nigra (SN) in monitored time interval post-HII.

#### Acute Metabolome Changes in the Neonatal Rat Brain under HI Insult

Neonatal rat brain rapidly increased levels of phosphocreatine (PCr) and amino acids in most regions of the IL hemisphere 12 h post-HII (Figure 1). Specifically, the PCr level was significantly increased in the anterior SCtx (1.5-fold, *p* = 0.024), MCtx (1.6-fold, *p* = 0.024), SN (1.5-fold, *p* = 0.0046), and Mid (1.5-fold, *p* = 0.0012). At the level of amino acids, the histidine level increased in anterior MCtx (1.8-fold, *p* = 0.015) and posterior SCtx [SCtx (P)] (1.7-fold, *p* = 0.0039) along with valine (1.7-fold, *p* = 0.0015) and glycine (1.7-fold, *p* = 0.0045). In the Hipp, the levels of all three amino acids increased, with histidine being more clear (1.8-fold, *p* = 0.0011) than valine (1.6-fold, *p* = 0.019) and glycine (1.4-fold, *p* = 0.045). Glycine and valine levels were more abundant in the Mid (1.3-fold, *p* = 0.0028 and 1.3-fold, *p* = 0.0092, respectively) and SN (1.3-fold, *p* = 0.0010 and 1.3-fold, *p* = 0.0275, respectively). In contrast, a decrease in proline levels was observed in the SCtx (P) of both the IL hemisphere (1.6-fold, *p* = 0.026) and contralateral (CL) hemisphere (1.4-fold, *p* = 0.0039) (Figure 1). This was one of a few cases, in which the CL hemisphere region was also affected. Later, at 24 and 36 h post-HII, PCr and amino acid levels returned to their control levels (Figures 1 and S2).

#### Subacute Changes in Brain Metabolome

In our model, the PCr/creatine (Cr) ratio varied among control brain regions, reflecting the variation in the energy metabolism activities. At 12 h post-HII, the PCr/Cr ratio in the IL hemisphere was 1.7-fold higher than that in sham brains. However, at 36 h post-HII, the PCr/Cr ratio was 1.8-fold lower in the HI-affected regions, particularly the CPu, accumbens nucleus core (AcbC), and anterior SCtx and MCtx (Table S1), due to a combination of PCr depletion and Cr elevation.

**Figure 3:**
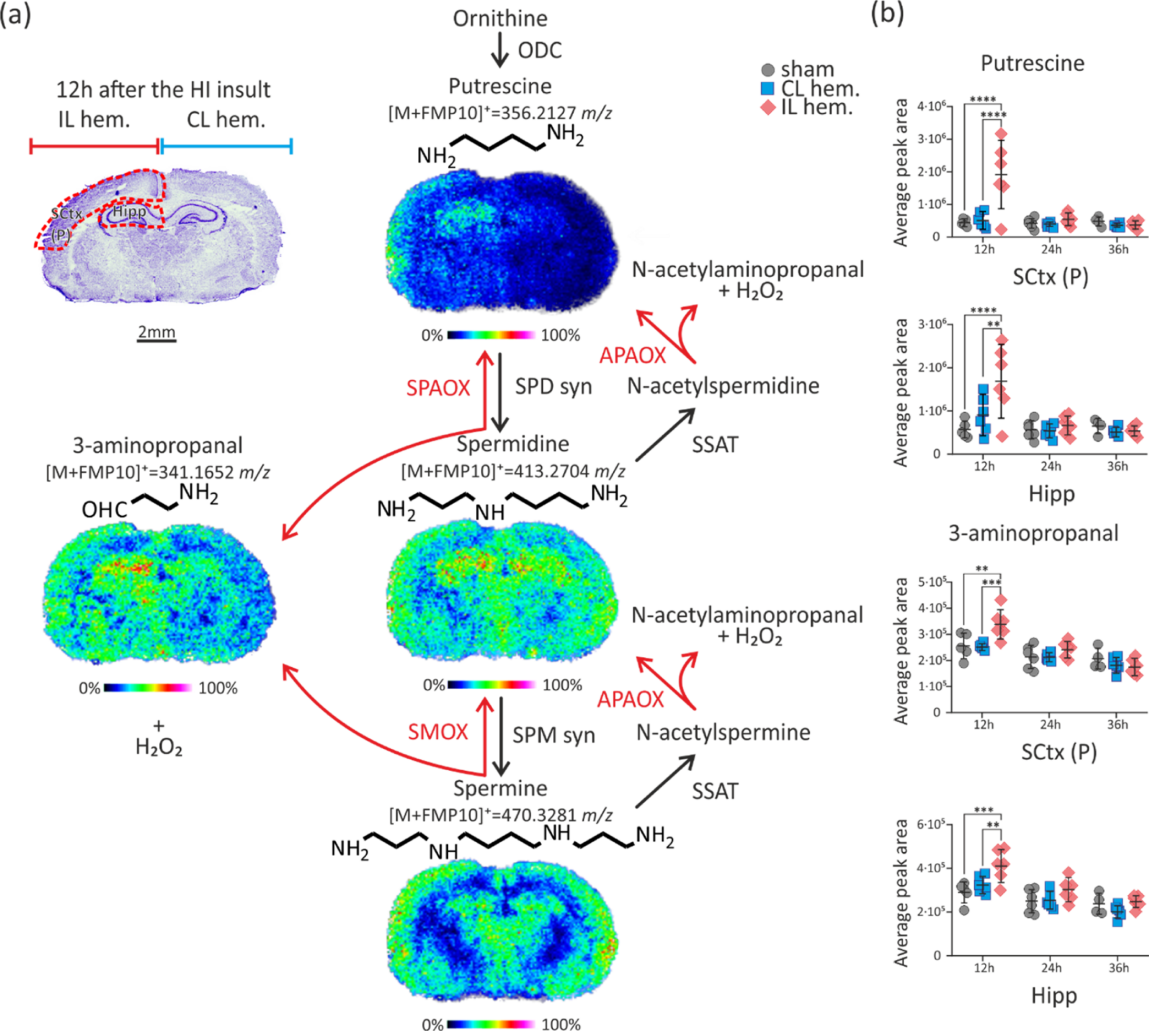
Disruption of the polyamine metabolic pathway after hypoxic-ischemic insult. (a) Nissl-stained brain section with MSI images of a monitored and enzymatically controlled polyamine metabolism pathway. Data were collected with 100 μm lateral step size and normalized to GABA-*d*_6_ internal standard. (b) Graphs showing the relative intensity levels of putrescine and 3-aminopropanal in the SCtx (P) and Hipp of control (sham) and HI-insulted animals 12, 24, and 36 h postinsult. Results of two-way ANOVA with Tukey’s multiple comparisons post hoc test (*n* = 4–6 biological replicates; for details, see the Materials and Methods section). Error bars indicate standard deviations: **p ≤ 0.01, ***p ≤ 0.001, and ****p ≤ 0.0001. Abbreviations: IL hem., ipsilateral hemisphere; CL hem., contralateral hemisphere; APAOX, acetylpolyamine oxidase; ODC, ornithine decarboxylase; SCtx (P), posterior somatosensory cortex; SMOX, spermine oxidase; SPD syn, spermidine synthase; SPM syn, spermine synthase; SSAT, spermidine/spermine N1-acetyltransferase 1; Hipp, hippocampus.

**Figure 4:**
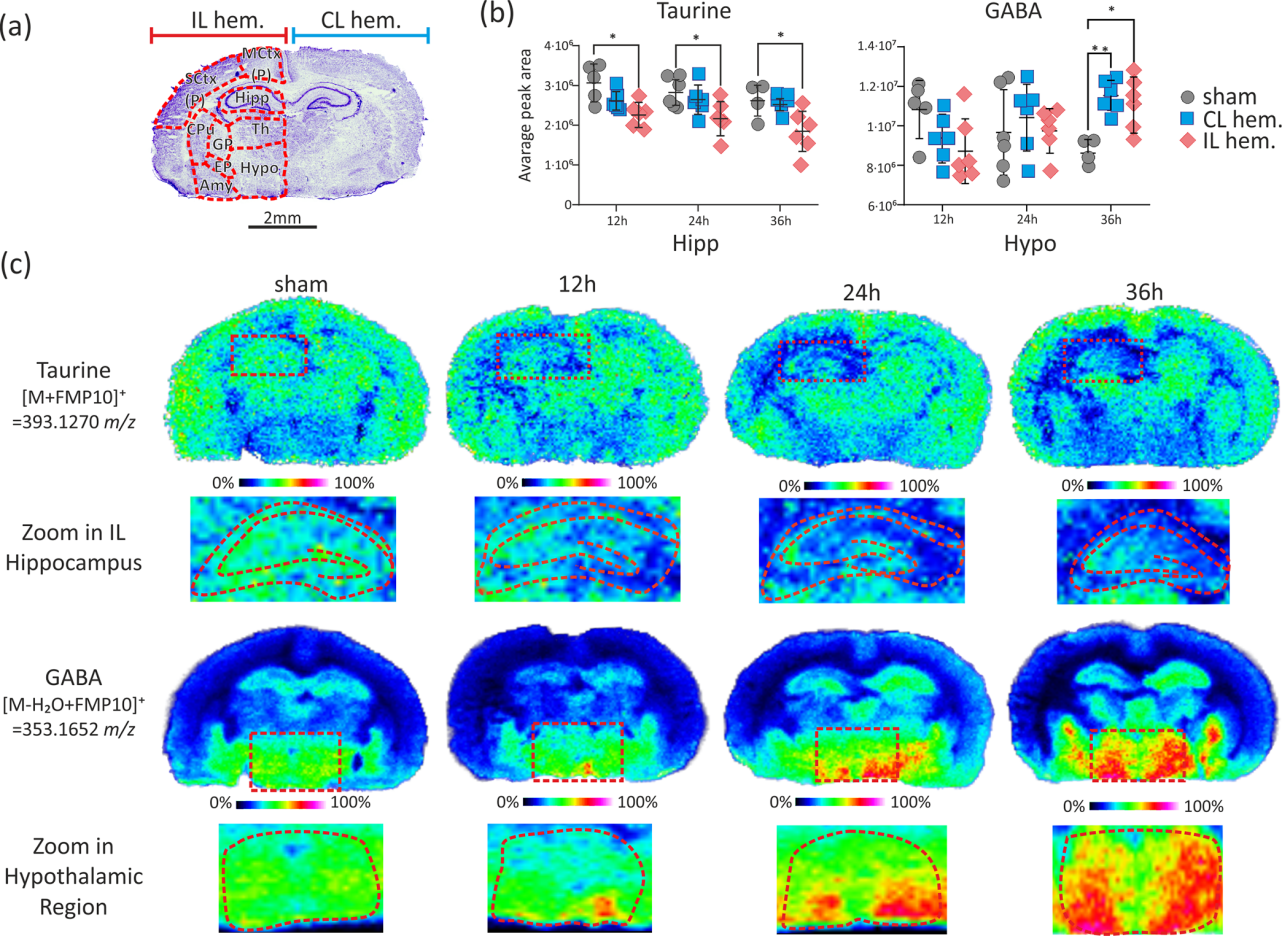
Time-resolved distributions of taurine and GABA in the neonatal rat brain after HI insult. (a) Nissl-stained brain section with specific regions depicted. (b) Graphs showing the relative intensity levels and statistics of taurine in the Hipp and GABA in the Hypo. Results of two-way ANOVA with Tukey’s multiple comparison post hoc test (*n* = 4–6 biological replicates; for details, see the Materials and Methods section) are shown. Error bars indicate standard deviations: *p ≤ 0.05, **p ≤ 0.01. (c) MALDI-MSI images of taurine and GABA distributions 12, 24, and 36 h post-HI insult and in a control (sham) brain with the zoomed regions of interest. Data were collected with a 100 μm lateral step size and normalized to the GABA-*d*_6_ internal standard. Abbreviations: IL hem., ipsilateral hemisphere; CL hem., contralateral hemisphere; Hypo, hypothalamic region; GP, globus pallidus; Th, thalamic region; Amy, amygdala; MCtx (P), posterior motor cortex; EP, entopeduncular nucleus; SCtx (P), posterior somatosensory cortex; CPu, caudate putamen; Hipp, hippocampus; GABA, γ-aminobutyric acid.

The polyamine system and metabolism of nonproteinogenic taurine and GABA were found to respond in the early stage of HI insult, and these changes lasted up to 36 h post-HII. While the levels of the higher polyamines spermine and spermidine remained constant in HI-affected regions, the levels of the lower polyamines (putrescine, 3-aminopropanal, and cadaverine) increased by 12 h dramatically (Figure 3). Specifically, they rose in cortex subregions, particularly SCtx (P) (putrescine, 4.3-fold, *p* = 0.0001; cadaverine, 6.2-fold, *p* = 0.0001; 3-aminopropanal, 1.3-fold, *p* = 0.0019), SN (putrescine, 2.1-fold, *p* = 0.0002; cadaverine, 3.0-fold, *p* = 0.0007; 3-aminopropanal, 1.5-fold, *p* = 0.0005), Hipp (putrescine, 3.0-fold, *p* = 0.0001; cadaverine, 3.9-fold, *p* = 0.0003; 3-aminopropanal, 1.4-fold, *p* = 0.0005), and Amy (putrescine, 3.3-fold, *p* = 0.0001; cadaverine, 5.1-fold, *p* = 0.0001; 3-aminopropanal, 1.1-fold, *p* = 0.3855). At 24 h post-HII, putrescine and cadaverine levels were substantially increased in SN (1.9-fold, *p* = 0.0142, and 2.4-fold, *p* = 0.0411, respectively) and Mid (1.8-fold, *p* = 0.0408, and 3.0-fold, *p* = 0.0074, respectively). Levels of lysine, taurine, and GABA were significantly affected by HI insult, too. Lysine levels were elevated in the IL hemisphere relative to control levels, peaking at 24 h post-HII, specifically in the Hipp (2.2-fold, *p* = 0.0321) and Mid (1.5-fold, *p* = 0.0148), while taurine levels were reduced at the 12, 24, and 36 h time points: 1.4-fold (*p* = 0.0112), 1.3-fold (*p* = 0.0386), and 1.4-fold (*p* = 0.0187), respectively (Figure 1). Levels of the GABA neurotransmitter varied with time in a region-specific manner in both the IL and CL hemispheres (Figure 4). HI insult resulted in a decrease in GABA levels at 12 h in the IL hemisphere, particularly in the SCtx (P) (1.5-fold, *p* = 0.0075), globus pallidus (GP, 2.0-fold, *p* = 0.0178), and Amy (1.4-fold, *p* = 0.0450). At 24 h, the GABA level returned to its control value but then increased at 36 h in the hypothalamic region (Hypo) (IL hemisphere, 1.3-fold, *p* = 0.0285; CL hemisphere, 1.3-fold, *p* = 0.0088) relative to controls. GABA levels also significantly increased in the CL hemisphere, particularly in the Hypo (1.2-fold from 12 to 36 h, *p* = 0.035) and GP (1.6-fold from 24 to 36 h, *p* = 0.043) (Figure S2). Of note, the increase in GABA level was exclusive to the CL hemisphere at the 36 h sampling time.

#### Delayed Metabolomic Alterations of the Neonatal Rat Brain

The basal ganglia were found to be severely affected by HI insult at both the molecular and cellular levels (Figures 1 and 2), indicating a particular susceptibility of this region to HI. Analysis of time-resolved changes in α-tocopherol levels revealed a substantial increase from 12 to 36 h post-HII, particularly in GP (1.6-fold, *p* = 0.021) and Hipp (1.4-fold, *p* = 0.039) (Figure S2). Norepinephrine and epinephrine levels significantly increased in the striatum subregions (Figure S3). At 24 h post-HII, the norepinephrine level was also markedly increased, particularly in the CPu of the IL hemisphere (1.5-fold, *p* = 0.010). Notably, its abundance in the dorsal CPu was associated with signs of severe neurodegeneration, including apoptosis and neuronal debris (Figure S3). Levels of epinephrine increased in CPu (1.8-fold, *p* = 0.019) and AcbC (2.1-fold, *p* = 0.044). At 36 h post-HII, higher dopamine levels were observed in the entopeduncular nucleus (EP, 1.5-fold, *p* = 0.0172).

## Discussion

**Figure 5:**
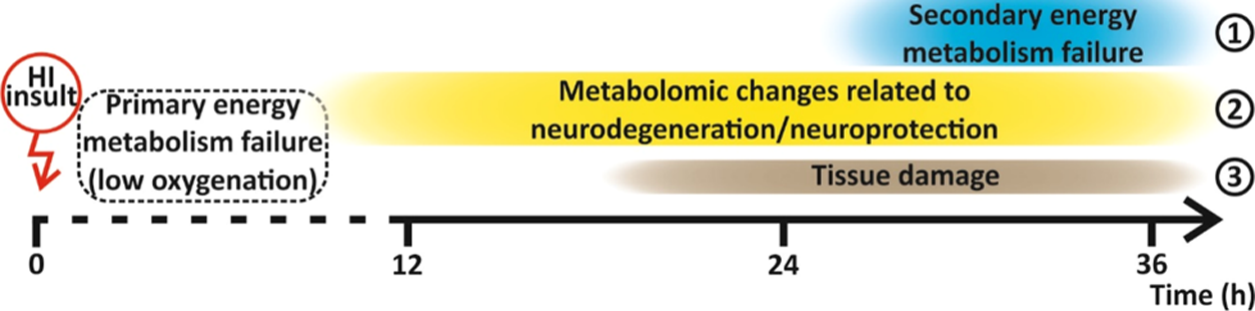
Time course of pathophysiological processes in the neonatal rat brain due to hypoxic-ischemic (HI) insult at the (1) molecular/cellular, (2) molecular, and (3) cellular/tissue levels.

The susceptibility of the immature brain to hypoxia–ischemia mainly depends on the temporal and regional status of critical developmental processes and brain metabolism. Our study defined region-specific early metabolic alterations accompanying HI insult that are rapidly changing within 36 h post-HII (Figure 5). The severity of delayed (secondary) energy metabolism failure in the brain largely determines neonatal outcome after a hypoxic-ischemic event. We showed that polyamine oxidation and region-specific dysregulation of the excitatory neurotransmitters glycine and GABA, as well as taurine and norepinephrine, play a critical role in oxidative brain damage in 7-day-old rat pups. In addition, we demonstrated that the extent of neuronal damage associates well with a regional and subregional altered occurrence of polyamines and neurotransmitters, which may predict the regional vulnerability of the brain to HII.

Delayed energy metabolism failure in the brain is associated with oxidative stress, excitotoxicity, and inflammatory processes, leading to cell death. In hypoxia and ischemia, reactive oxygen species (ROS) are reported to be generated mainly by NADPH oxidases, uncoupled nitric oxide synthase, and oxygen-depleted mitochondrial electron transport.^24,25^ In this study, we demonstrate the oxidation of polyamines as another source of H_2_O_2_ production and cell toxicity that mediates HI-induced cell death. Polyamine levels are tightly regulated at the level of synthesis, transport, and degradation by a series of enzymatically controlled reactions.^26^ We report significantly elevated levels of putrescine at 12 h post-HII in all monitored brain regions, with sustained increases selectively in EP, SN, and Mid at 24 h post-HII. On the one hand, the results indicate increased activity of ornithine decarboxylase (ODC), a rate-limiting enzyme in polyamine biosynthesis, which has region-specific activity expression^27^ and unspecifically converts lysine into cadaverine,^28^ which colocalized with putrescine. However, increased ODC activity has been associated with both neuroprotective^29,30^ and neurodegenerative^27,31^ effects in cerebral ischemia. On the other hand, in our experiment, a high regional putrescine level was positively correlated with significantly increased abundance of 3-aminopropanal. This result implies the overactivation of polyamine oxidation from spermine back to putrescine involved in a polyamine interconversion pathway via the action of spermine oxidase and serum polyamine oxidase.^32^ The catabolic pathway produces highly toxic byproducts. In particular, cytoplasmic and nuclear-localized spermine oxidase converts spermine to spermidine by producing substantial amounts of H_2_O_2_ and a reactive aldehyde, 3-aminopropanal, which spontaneously converts to acrolein.^26,32^ In addition, 3-aminopropanal is reported to be a potent lysosomal neurotoxin in cerebral ischemia, mediating progressive neuronal necrosis and glial apoptosis.^33,34^

Excitotoxicity is a key mediator of neuronal loss resulting from HII. Excessive levels of glutamate in a synaptic cleft, together with membrane depolarization, contribute to the opening of *N*-methyl-d-aspartate (NMDA), a subtype of the glutamate receptor, and α-amino-3-hydroxy-5-methyl-isoazole-4-propionic acid receptors, triggering intracellular Ca^2+^ overload and sending cells into a death spiral.^35^ In addition to glutamate,^23^ we showed that the abundance of an excitatory neurotransmitter, glycine, was remarkably high at 12 h post-HII and dominated in SCtx, SN, Hipp, and Mid, which showed signs of neuronal cell death. Glycine coactivates NMDA receptors and preferentially binds to extrasynaptic NMDA receptors, which have been associated with receptor-mediated calcium influx and neurotoxicity.^36−39^ In addition, MALDI-MSI revealed transiently elevated levels of norepinephrine together with epinephrine, mainly in CPu, which was associated with the onset of neuronal apoptosis-like cell death. While low levels of norepinephrine in rat brain synaptosomes protect the brain from oxidative insult by reducing lipid peroxidation and ROS, high levels induce cell death,^40^ which would be consistent with our results. As previously reported, NMDA receptors mediate glutamate-stimulated norepinephrine release,^39^ which provoked apoptosis of neonatal rat heart cardiomyocytes through ROS.^41^ In ischemia, high levels of norepinephrine led to epinephrine synthesis through phenylethanolamine-*N*-methyltransferase activity.^42^

In contrast to glycine and norepinephrine, we reported a decrease in GABA levels in critically affected SCtx, GP, Amy, and Hipp, 12 h after HII. This may prevent the exacerbation of HI injury by reducing the intracellular accumulation of Ca^2+^.^43,44^ Consistent with our study, Jiang et al.^45^ and Anju et al.^46^ reported that HII significantly diminishes the level of GABA in the neonatal rat brain. As a result of the inhibition of glutamic acid decarboxylase activity due to acute hypoxia,^47^ low levels of GABA consequently downregulate GABA receptor activity,^46^ which normally allows the passage of chloride ion efflux, resulting in depolarizing response. In fact, a potential neuroprotective brain strategy mediated by the reported low GABA early after the HII may further imbalance activity of the GABAergic system, which accounts for respiratory inhibition,^48^ neonatal seizures, and epilepsy^49^ later in life. Furthermore, HI-induced ion pump disturbance leading to cellular edema and impaired neurotransmission can be consequently related with a persistently decreased low level of taurine up to 36 h post-HII that agree with previously reported studies using magnetic resonance spectroscopy.^17,50^

Contrary to MALDI-MSI analysis, which provided 22 characteristic compounds, only eight of them were detected in the CSF: 5-HIAA, 3-aminopropanal, lysine, histidine, proline, GABA, α-tocopherol, and creatinine. We found no significant correlations between their levels and differences between control and HI-insulted brains at any sampling time (Figure S4). The sensitivity loss can be explained by dilution and reabsorption of specific metabolites from the CSF, *i.e.*, the fluid reflecting the bulk status of the whole brain volume.

In summary, the present study showed the capacity of MALDI-MSI providing comprehensive spatial neurometabolomics in the neonatal brain. Particularly, the results demonstrate unknown molecular alterations in polyamines and neurotransmitters involved in delayed secondary metabolism brain failure due to HII in 7-day-old rat pups that colocalized with neurodegenerative signs of cellular death. To minimize nonspecific effects due to sampling procedures, including CSF collection, decapitation, and skull removal, which might alter the HI-induced changes in the levels of molecules of interest,^51^ the complete samplings were performed under isoflurane anesthesia in less than 2 min. This brief period is deemed safe for metabolomic studies in the mouse brain.^52^ It is also noteworthy that the same procedures were applied to the group of sham animals, in which HI-specific biomarker molecular levels were assessed. Although a certain correlation between the molecular changes and cellular and tissue alterations was observed, a conceivable causal relationship needs to be proved. On the contrary, the targeted prevention of excessive polyamine oxidation and protection of energy metabolism and antioxidant pool in the neonatal brain under HI insult may be considered in further studies, aiming to provide better outcomes of neonatal hypoxic-ischemic encephalopathy.

## Materials and Methods

### Materials

Isoflurane anesthetic and nitrogen gas of 4.0 purity were obtained from Baxter and Linde (Bratislava, Slovak Republic), respectively. Deuterium-labeled standards, including γ-aminobutyric acid (GABA)-*d*_6_, homovanillic acid (HVA)-*d*_5_, 5-hydroxytryptamine (5-HT)-*d*_4_, and dopamine (DA)-*d*_4_, were purchased from Qmx Laboratories (Thaxted, U.K.). The FMP-10 matrix was purchased from Tag-ON (Uppsala, Sweden). 5-Hydroxyindoleacetic acid (5-HIAA), 5-HT, dopamine, 3-methoxytyrosine (3-MT), norepinephrine, epinephrine, GABA, putrescine, proline, taurine, spermidine, histidine, glycine, valine, lysine, and spermine standards used for MS/MS analysis were purchased from Sigma-Aldrich (Stockholm, Sweden). Liquid chromatography-MS (LC-MS) grade acetonitrile, methanol, and water were purchased from Merck (Solna, Sweden), Sigma-Aldrich (Prague, Czech Republic), and Honeywell (Prague, Czech Republic). Indium tin oxide (ITO) glass slides for MALDI-MSI were purchased from Bruker Daltonics (Bremen, Germany).

### Animal Model

All animal manipulation was conducted in accordance with European Directive 2010/63/EU and guidelines of the Slovak Animal Protection Act (Directive No. 377/2012 and Regulation No. 436/2012). In compliance with the ARRIVE guidelines,^53^ prior approval was obtained from the State Veterinary and Food Administration of the Slovak Republic (Decision No. 2575/11–221/3) and the Animal Welfare Committee of the Institute of Experimental Pharmacology and Toxicology, Centre of Experimental Medicine of the Slovak Academy of Sciences in Bratislava. Wistar rat females were mated with males (both from the Breeding Station in Dobra Voda, Trnava District, Slovak Republic) at a 3:1 ratio for 6 days, placed in separate cages, and allowed to give birth at term. Seven-day-old male pups (13 to 18 g) were used for all experiments. The corresponding brain maturation is close to the developmental degree of near-term human infants (at 32–36 weeks of gestation) highly susceptible to cerebral HI injury.^54^ Notably, males were selected due to their higher susceptibility to HI insult than females with more pronounced brain damage.^55^

The rat animal model of HI was generated following the Rice–Vannucci protocol,^12^ with adaptation, as follows. Under general anesthesia with isoflurane (4% for induction and 2% for maintenance), the left common carotid artery of pups was ligated with surgical silk. After closing the neck incision, the pups were allowed to recover for 1 h under the care of their dams and subsequently exposed to hypoxic conditions (8% oxygen), obtained by mixing air with nitrogen gas, in a temperature-controlled sealed chamber at 34 °C for 90 min. A slightly lower temperature prevents previously observed complete degeneration and loss of highly susceptible brain regions at 37 °C,^56,57^ thus allowing relevant metabolomic and cellular analysis across the HI-injured neonatal rat brains without compromising neuronal degeneration.^3,16^ As controls, sham animals were subjected to anesthesia, a surgical neck incision, and common left carotid artery isolation without ligation. After 1 h of recuperation, sham pups were placed in a temperature-controlled sealed chamber at 34 °C for 90 min in normoxia. Brain samples were obtained at 12, 24, and 36 h after the onset of the insult, including HI-affected (*n* = 6 for each time interval) and sham controls (*n* = 6 for each time interval). The minimally required per-group sample size for a two-sided test was estimated, given the probability level (α = 0.05), the anticipated effect size (Cohen’s *d* = 1.8), and the desired statistical power level (0.8).^58^ Before brain dissection, CSF was collected according to a previously published protocol.^59^ A direct cisterna magna puncture with a 0.5 mm (o.d.) needle was performed under isoflurane anesthesia (<2 min), which is more recommended for metabolomic studies than euthanasia^60^ and had an insignificant impact on neurotransmitters and related metabolites. CSF was allowed to drain freely into the needle and then picked up by a micropipette. CSF was centrifuged at 7000 rpm for one min to avoid cell contamination and stored in vials at −80 °C until analysis. After the CSF sampling was finished, the rat pup was sacrificed by decapitation; its brain was quickly removed from the skull, snap-frozen in liquid nitrogen, and stored at −80 °C.

### Sample Preparation

Before cryosectioning, deeply frozen brain samples were allowed to warm at −12 °C in a Leica CM1900 cryostat chamber (Wetzlar, Germany) for 1 h. Three levels of each brain—striatal (bregma +2.28 mm), hippocampal (bregma −2.92 mm), and substantia nigra (bregma −6.00 mm)—were cryosectioned according to the neonatal and adult stereotaxic atlases.^61,62^ Consecutive 12-μm-thick brain sections, used for MALDI-MSI of neurotransmitters and metabolites,^20^ were thaw-mounted onto precooled ITO and microscopic glass slides for mass spectrometric and microscopic analysis, respectively. The optimum brain section thickness was chosen according to a study by Vonnie et al.^63^ The slides were then quickly dried with N_2_ gas and stored at −80 °C. On the analysis day, sections were dried under a flow of N_2_ and vacuum-desiccated for 60 min before and between internal standards and matrix spraying. A mixture of deuterium-labeled standards, including GABA-*d*_6_, 5-HT-*d*_4_, HVA-*d*_5_, and DA-*d*_4_ at 33.3, 0.05, 0.3, and 0.1 μg/mL, respectively, in 70% methanol was sprayed over tissue sections using a TM-Sprayer (HTX-Technologies). The spraying parameters were as follows: temperature, 90 °C; flow rate, 0.07 mL/min; nozzle velocity, 1100 mm/min; track spacing, 2.0 mm; N_2_ pressure, 6 psi; number of passes, 6; spraying pattern, horizontal/vertical. Next, the samples were subjected to on-tissue derivatization according to a previously published protocol.^20^ Briefly, FMP-10 solution (1.8 mg/mL) in 70% acetonitrile was sprayed over the tissue sections with the TM-Sprayer and identical parameters except for flow rate, 0.08 mL/min; number of passes, 20; and spraying pattern, horizontal.

Deeply frozen CSF samples were analyzed with an adapted protocol.^64^ The CSF sample (0.5 μL) was mixed with an internal standard, 0.2 μL of GABA-*d*_6_ in 50% MeOH (1.66 μg/mL), and the whole volume (0.7 μL) was spotted onto a MALDI ground steel target plate (Bruker Daltonics, Germany) and allowed to dry. The FMP-10 derivatization agent was then sprayed over the target plate with the same parameters as those used for brain sections, except that the number of passes was 10. Before MALDI-MSI analysis, the MALDI slide adapter with prepared glass slides and ground steel target plate was scanned using a flatbed optical scanner (Epson Perfection V500, Japan).

### MSI Data Acquisition

All MSI tissue experiments were carried out using a solariX 7T-2Ω MALDI Fourier transform ion cyclotron resonance (FTICR) MS instrument (Bruker Daltonics, MA) equipped with a Smartbeam II 2 kHz laser. Data were acquired in positive ion mode using quadrature phase detection (2 Ω) in a mass range of 150 to 1000 *m*/*z* calibrated against red phosphorus clusters before analysis. The ion signal at *m*/*z* 555.2231 (FMP-10 ion cluster) was used for the online data calibration. The time domain file size was set to 2 M, and ion optics was optimized to collision cell voltage (−1.5 V, DC bias: 0.7 V), time-of-flight delay (0.75 ms), and transfer optics (4 MHz, Q1 *m*/*z* 379). The laser power was tuned at the beginning of the experiment, and the parameters were kept constant throughout each MALDI-MSI data acquisition (200 laser shots/position). All MSI data were acquired with 100 μm lateral resolution. CSF samples were analyzed with similar instrumental parameters using a solariX 12T-2Ω MALDI FTICR instrument (Bruker Daltonics, MA). The standard of metabolites was dissolved in 50% aqueous methanol or pure water (1 μg/mL), depending on the compound’s solubility. The standard solutions (1 μL) were spotted on the ITO glass slide and overlaid with the FMP-10 matrix in a homogeneous manner analogous to tissue analysis. On-glass (standards) and on-tissue (analytes) product ion mass spectra of compounds of interest were acquired with compound-specific collision energy and compared (Figure S5).

### MSI Data Processing

Images of distributions of detected metabolites were generated with FlexImaging software (Bruker Daltonics, Germany, v.5.0), in which the annotation of regions of interest in sham and HI-insulted brains using stereotaxic atlases^61,62^ was performed. Data related to the two hemispheres of HI insult brains were separately processed. This is because the IL hemisphere (side of the left common carotid artery ligation) was fully HI-insulted tissue, while the CL (right) hemisphere was regarded as only affected by hypoxia due to the preservation of the blood supply via the right common carotid artery. MSI data were processed in SCiLS Lab software (Bruker Daltonics, Germany, v.2019c Pro). For the relative quantitation of detected metabolites, MSI data were normalized to the corresponding deuterated internal standard or root-mean-squared values (Table S2). The FMP-10 molecule can be identified in three distinct molecular forms, each attached to either a primary amine or a phenolic hydroxyl group on a target molecule. This results in multiple *m*/*z* values for each target molecule. For molecules with a single reactive site, derivatization with FMP-10 introduces a positive charge (*z* = +1). Molecules with more than one reactive site can form singly derivatized (M + [FMP-10]) or doubly derivatized (M + 2[FMP-10]-H+ or M + 2[FMP-10]-CH_3_) complexes (*z* = +1).^20,65^ During the data analysis phase, all derivatized signals were considered. By evaluating the overlapping signals and the signal-to-noise ratios of the derivatized ions, only the most intense and interference-free signals were utilized for data evaluation.

For each analyte, the average area under the curve within the region of interest was used for data extrapolation. Brain energy metabolism was assessed using the PCr to Cr ratio. While reductions in PCr levels reportedly reflect a failure of oxidative phosphorylation and adenosine triphosphate production,^66^ the PCr/Cr ratio can be used to indicate brain energy status.^67^

### Statistics

Exported data were statistically processed using GraphPad Prism v.8.4 (GraphPad Software, CA) and OriginPro 2022 (OriginLab Corporation) software. Six pups were included in the sham and experimental groups. Two complete brains from the sham group (12 and 36 h) were excluded due to mechanical damage during brain removal. In addition, hippocampal and striatal levels in a single sham animal (36 h) were used for method development. The normality of distributions of analyte levels within groups of samples was assessed with Shapiro–Wilk and Kolmogorov–Smirnov tests. Outliers, *i.e.*, anterior MCtx and SCtx of one sham at 24 h and CPu and AcbC of one HI-affected brain at 36 h, were identified by the ROUT method with *Q* = 1% and excluded from further analyses. In summary, four to six biological brain replicates were used for the data evaluation. For data meeting normality of distribution criteria, two-way analysis of variance (ANOVA) with Tukey’s multiple comparison post hoc test was applied to compare metabolomes of specific brain regions sampled at different time points (12, 24, or 36 h postinsult) and between corresponding insulted (IL and CL) and sham samples. Kruskal–Wallis ANOVA with Dunn’s multiple comparison test was used for nonparametrically distributed data. If two out of the three insulted (IL and CL) and sham groups or time-based (12, 24, and 36 h after insult onset) groups had to be processed with Kruskal–Wallis ANOVA, one-way ANOVA with Tukey’s multiple comparisons post hoc test was used for the third group. Instrumental reproducibility was assessed by examining data recorded for three consecutive tissue slices and CSF sample replicates providing the coefficients of variance mostly below 15% (Table S3).

### Histology

MSI-processed and consecutive brain sections were subjected to Nissl staining to examine and visualize neuronal tissue. Before being stained, the FMP-10 matrix was removed in 100% EtOH for 1 min. Then, the tissue sections were fixed with absolute EtOH for 5 min and allowed to dry for 5 min. After a subsequent wash with water for 1 min, tissues were stained with cresyl violet solution (5 mg/mL in 0.2% acetic acid) for 12 min, followed by a 5 min wash with water and dehydration by serial incubation in 90% EtOH and 95% EtOH for 3 min and then 100% EtOH for 6 min. The tissue sections were dried again for 5 min and cleared in xylene for 10 min. The glass slides were covered with dibutylphthalate-polystyrene-xylene mounting medium and cover glass; then the tissue sections were examined by optical microscopy to assess and compare morphological markers on sections of HI-insulted brain IL and CL hemispheres and sham brains.

## Data Availability

The MALDI–MSI data sets supporting this study are available from the corresponding author (VH) upon request. MS and MS/MS spectra for derivatized standards and analytes are available in the Supporting Information.

## Supporting Information Available

Experimental workflow of neonatal rat brain HI insult assessment by MALDI-MSI and histological examination; time courses of relative intensity levels of compounds detected in indicated brain regions; intraregional increases in norepinephrine levels related to neurodegeneration in the neonatal rat brain after hypoxic-ischemic (HI) insult; time courses of relative intensity levels of the compounds detected in the cerebrospinal fluid; fragmentation spectra of the analyzed compounds; phosphocreatine (PCr)/creatine (Cr) ratio; normalizations used for all detected compounds; reproducibility table (PDF)

## Abbrevations

3-MT: 3-methoxytyramine
5-HIAA: 5-hydroxyindoleacetic acid
5-HT: 5-hydroxytryptamine
A: anterior
AcbC: accumbens nucleus core
ADP: adenosine diphosphate
AMP: adenosine monophosphate
Amy: amygdala
ANOVA: analysis of variance
APAOX: acetylpolyamine oxidase
CL: contralateral
CPu: caudate putamen
Cr: creatine
CSF: cerebrospinal fluid
DA: dopamine
EP: entopeduncular nucleus
FMP-10: 4-(anthracen-9-yl)-2-fluoro-1-methylpyridin-1-ium iodide
FTICR: Fourier transform ion cyclotron resonance
GABA: γ-aminobutyric acid
GP: globus pallidus
HI: hypoxic-ischemic
HII: hypoxic-ischemic insult
Hipp: hippocampus
HVA: homovanillic acid
Hypo: hypothalamic region
IL: ipsilateral
ITO: indium tin oxide
MALDI: matrix-assisted laser desorption ionization
MCtx: motor cortex
Mid: midbrain
MRI: magnetic resonance imaging
MRS: magnetic resonance spectroscopy
MSI: mass spectrometry imaging
NMDA: *N*-methyl-d-aspartate receptor
ODC: ornithine decarboxylase
P: posterior
PCr: phosphocreatine
ROS: reactive oxygen species
SCtx: somatosensory cortex
SD: standard deviation
SMOX: spermine oxidase
SN: substantia nigra
SPD syn: spermidine synthase
SPM syn: spermine synthase
SSAT: spermidine/spermine N1-acetyltransferase 1
Th: thalamic region
