# Supplementary material for: Hypoxic-Ischemic Insult Alters Polyamine and Neurotransmitter Abundance in the Specific Neonatal Rat Brain Subregions
Source: ACS Chem Neurosci. 2024 Jul 26;15(15):2811–21. doi: 10.1021/acschemneuro.4c00190 (PMC11311127; doi:10.1021/acschemneuro.4c00190)
Supplement: Supplementary file 1 — cn4c00190_si_001.pdf [file cn4c00190_si_001.pdf]

Supplementary material

Hypoxic-ischemic insult alters polyamine and neurotransmitter abundance in the specific neonatal rat brain subregions

Hynek Mácha[1,2], Dominika Luptáková[1,3,4], Ivo Juránek[5*], Per E. Andrén[3*], Vladimír Havlíček[1,2*]

[1] Institute of Microbiology of the Czech Academy of Sciences, Vídeňská 1083, Prague 142 00, Czech Republic

[2] Department of Analytical Chemistry, Faculty of Science, Palacký University, 17. listopadu 12, Olomouc 771 46, Czech Republic

[3] Department of Pharmaceutical Biosciences, Spatial Mass Spectrometry, Science for Life Laboratory, Uppsala University, Husargatan 3, Uppsala 75124, Sweden

[4] Biomedical Research Center, Slovak Academy of Sciences, Dubravska cesta 9, 845 05 Bratislava, Slovak Republic

[5] Centre of Experimental Medicine, Slovak Academy of Sciences, Dúbravská cesta 9, 841 04 Bratislava, Slovak Republic

* Corresponding authors: vlhavlic@biomed.cas.cz; per.andren@uu.se; ivo.juranek@savba.sk;

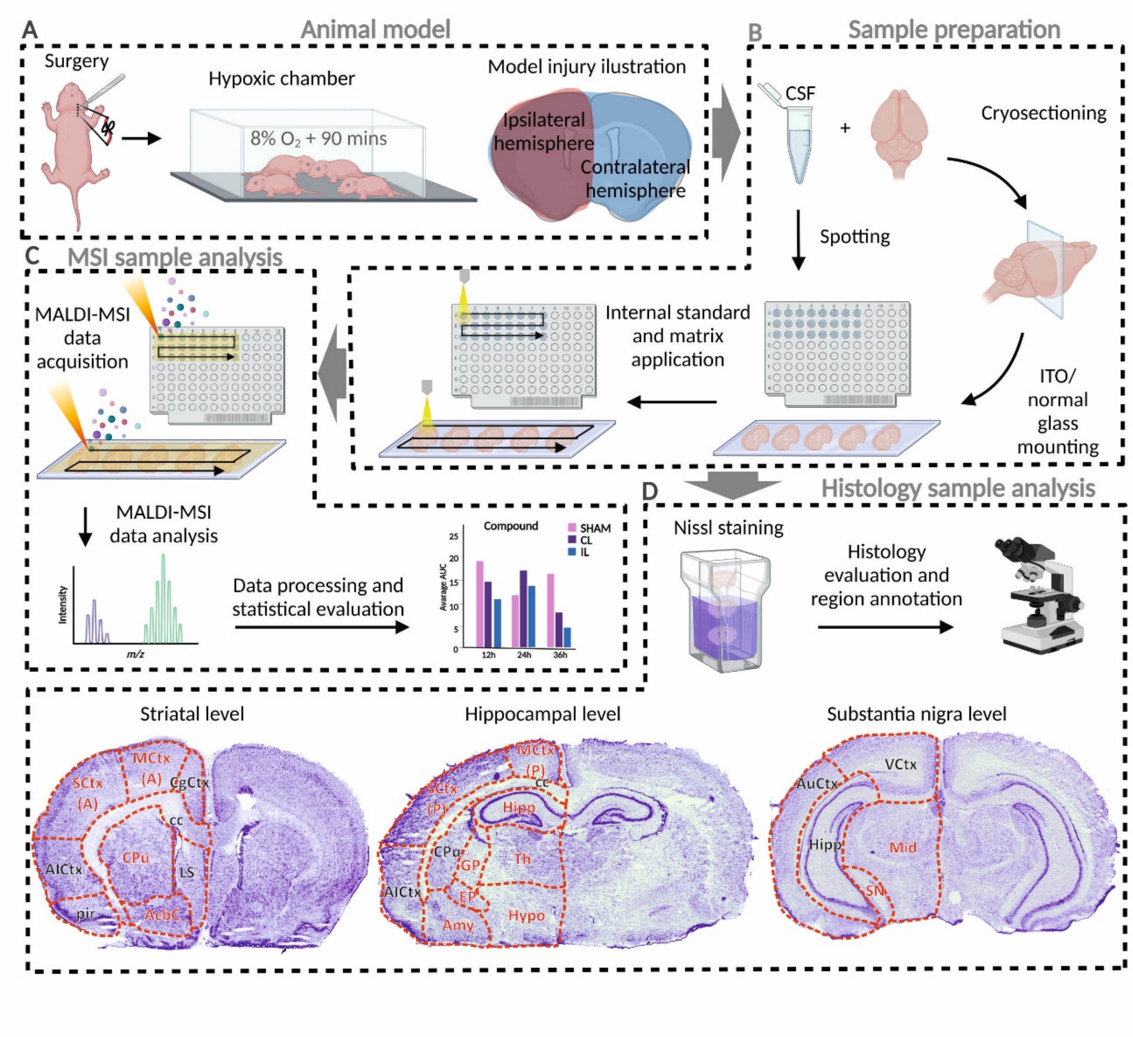

Figure S1. Experimental workflow of neonatal rat brain HI insult assessment by MALDI-MSI and histological examination. (A), animal model; **(B)**, sample preparation; **(C)**, sample analysis by mass spectrometry imaging; **(D)**, histological evaluation with region annotation. *Note*: brain areas marked in red were specifically processed and evaluated. Hypo, hypothalamic region; GP, globus pallidus; Th, thalamic region; Amy, amygdala; MCtx, motor cortex; EP, entopeduncular nucleus; SCtx, somatosensory cortex; CPu, caudate putamen; Hipp, hippocampus; cc, corpus callosum; pir, piriform cortex; LS, lateral septal nuclei; AcbC, accumbens nucleus core; CgCtx, cingulate cortex; AICtx, agranular insular cortex; AuCtx, auditory cortex; VCtx, visual cortex; SN, substantia nigra; Mid, midbrain; P, posterior; A, anterior; CL, contralateral hemisphere; IL, ipsilateral hemisphere; CSF, cerebrospinal fluid. (Created with BioRender.com)

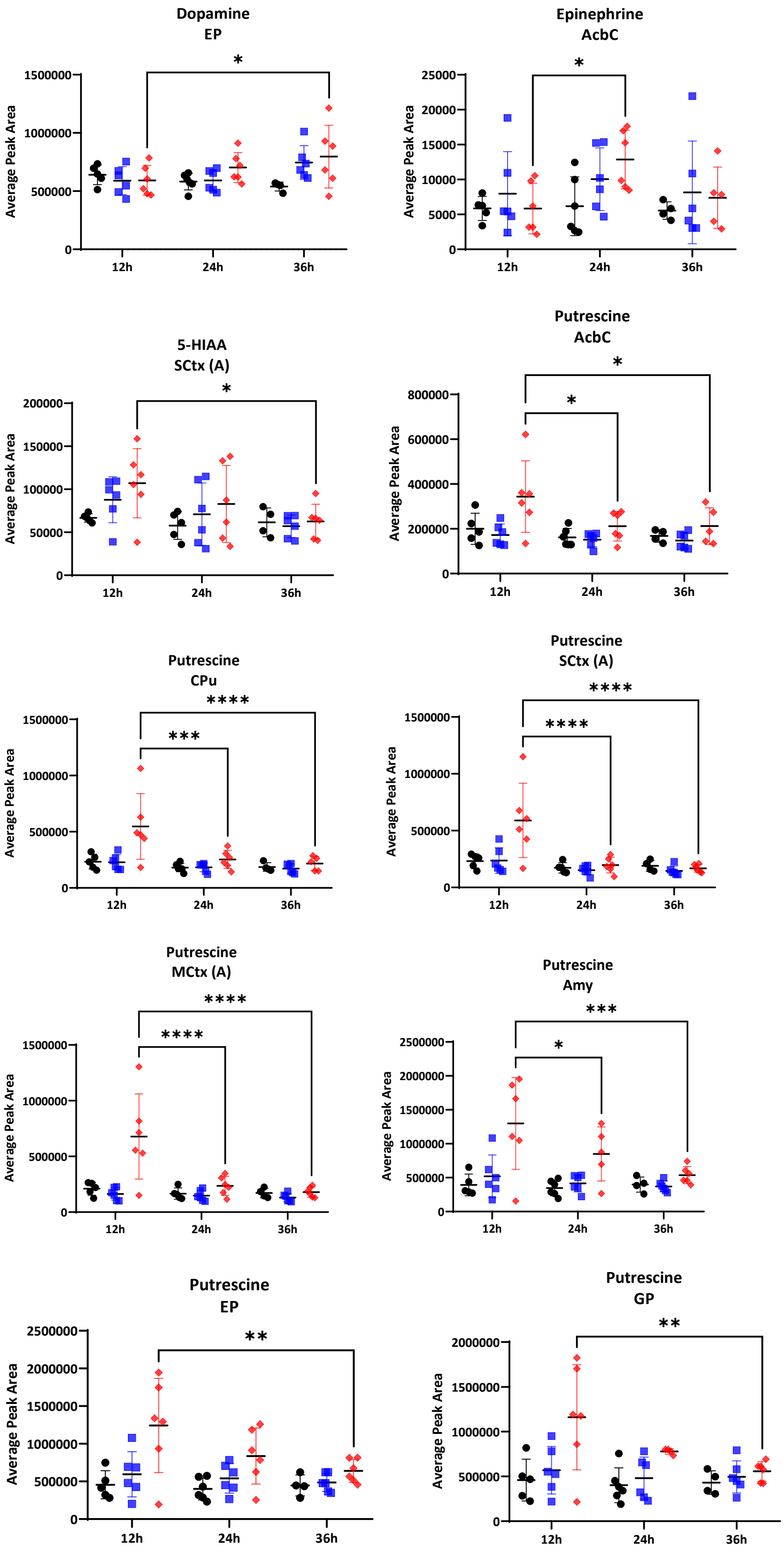
Dopamine
EP
*
Average Peak Area
1500000
1000000
500000
0
12h
24h
36h
Epinephrine
AcbC
*
Average Peak Area
25000
20000
15000
10000
5000
0
12h
24h
36h
5-HIAA
SCtx (A)
*
Average Peak Area
200000
150000
100000
50000
0
12h
24h
36h
Putrescine
AcbC
*
*
Average Peak Area
800000
600000
400000
200000
0
12h
24h
36h
Putrescine
CPu
****
***
Average Peak Area
1500000
1000000
500000
0
12h
24h
36h
Putrescine
SCtx (A)
****
****
Average Peak Area
1500000
1000000
500000
0
12h
24h
36h
Putrescine
MCtx (A)
****
****
Average Peak Area
1500000
1000000
500000
0
12h
24h
36h
Putrescine
Amy
***
*
Average Peak Area
2500000
2000000
1500000
1000000
500000
0
12h
24h
36h
Putrescine
EP
**
Average Peak Area
2500000
2000000
1500000
1000000
500000
0
12h
24h
36h
Putrescine
GP
**
Average Peak Area
2000000
1500000
1000000
500000
0
12h
24h
36h

S3

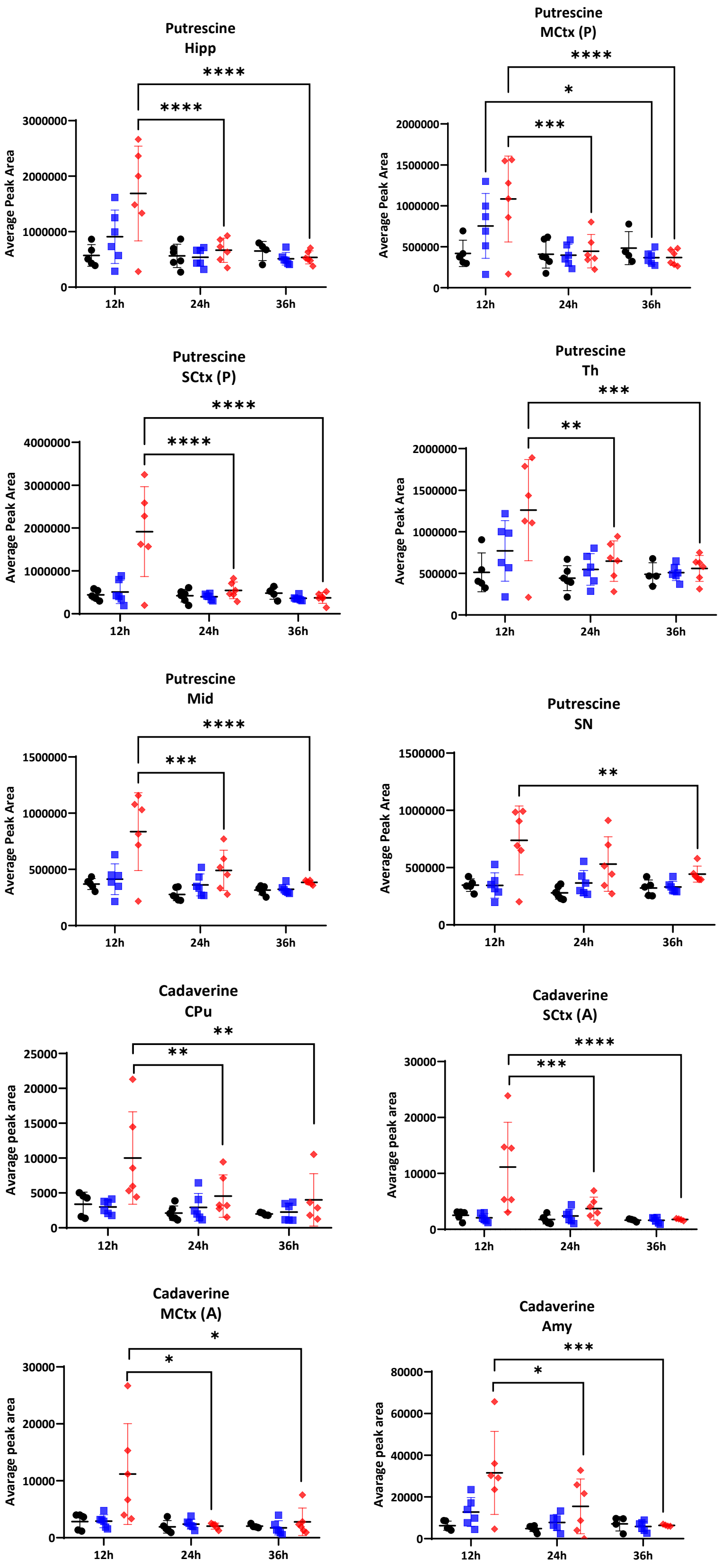
Putrescine
Hipp
Average Peak Area
3000000
2000000
1000000
0
12h
24h
36h
****
****
Putrescine
MCtx (P)
Average Peak Area
2000000
1500000
1000000
500000
0
12h
24h
36h
****
*
***
Putrescine
SCtx (P)
Average Peak Area
4000000
3000000
2000000
1000000
0
12h
24h
36h
****
****
Putrescine
Th
Average Peak Area
2000000
1500000
1000000
500000
0
12h
24h
36h
***
**
Putrescine
Mid
Average Peak Area
1500000
1000000
500000
0
12h
24h
36h
****
***
Putrescine
SN
Average Peak Area
1500000
1000000
500000
0
12h
24h
36h
**
Cadaverine
CPu
Avarage peak area
25000
20000
15000
10000
5000
0
12h
24h
36h
**
**
Cadaverine
SCtx (A)
Avarage peak area
30000
20000
10000
0
12h
24h
36h
****
***
Cadaverine
MCtx (A)
Avarage peak area
30000
20000
10000
0
12h
24h
36h
*
*
Cadaverine
Amy
Avarage peak area
80000
60000
40000
20000
0
12h
24h
36h
***
*

S4

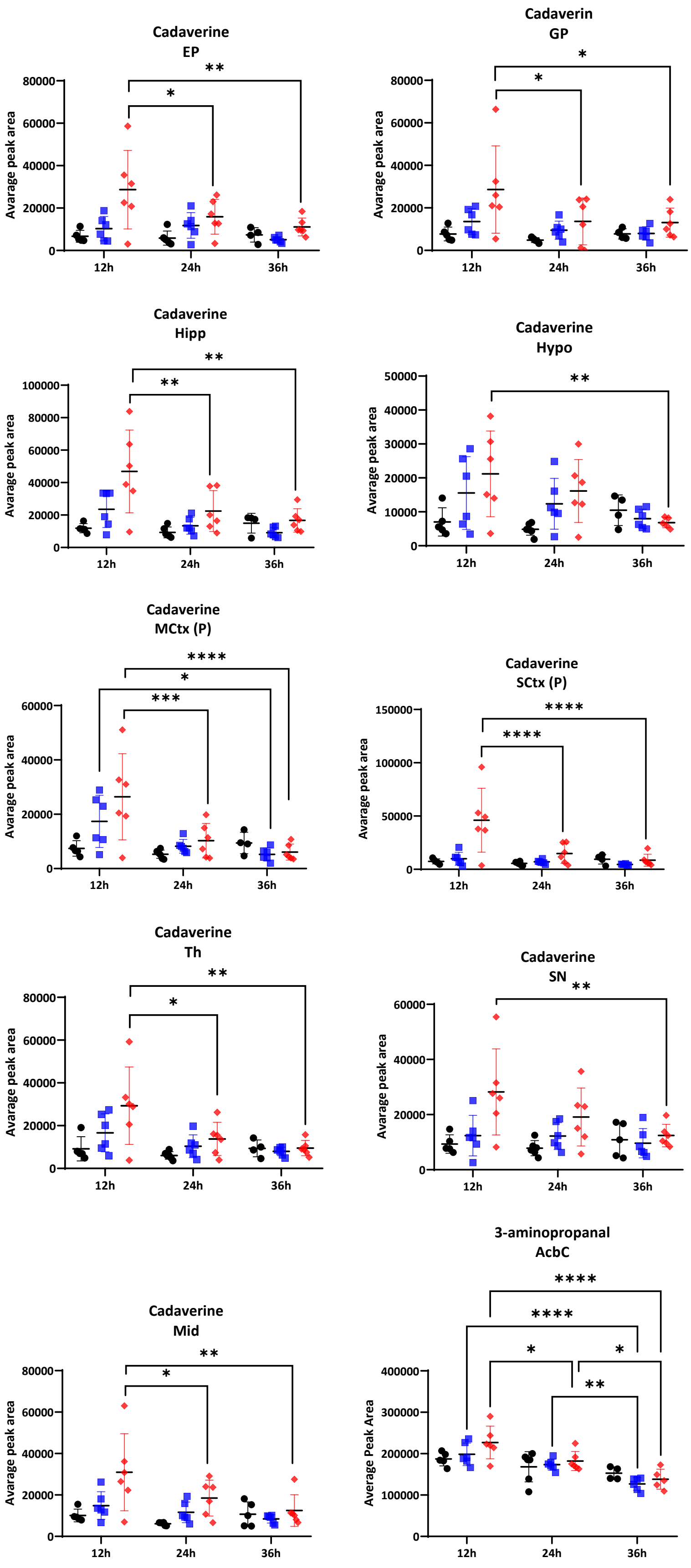

Cadaverine
EP
Avarage peak area
80000
60000
40000
20000
0
12h
24h
36h
**
*
Cadaverin
GP
Avarage peak area
80000
60000
40000
20000
0
12h
24h
36h
*
*
Cadaverine
Hipp
Avarage peak area
100000
80000
60000
40000
20000
0
12h
24h
36h
**
**
Cadaverine
Hypo
Avarage peak area
50000
40000
30000
20000
10000
0
12h
24h
36h
**
Cadaverine
MCtx (P)
Avarage peak area
60000
40000
20000
0
12h
24h
36h
****
*
***
Cadaverine
SCtx (P)
Avarage peak area
150000
100000
50000
0
12h
24h
36h
****
****
Cadaverine
Th
Avarage peak area
80000
60000
40000
20000
0
12h
24h
36h
**
*
Cadaverine
SN
Avarage peak area
60000
40000
20000
0
12h
24h
36h
**
3-aminopropanal
AcbC
Average Peak Area
400000
300000
200000
100000
0
12h
24h
36h
****
****
*
*
**
Cadaverine
Mid
Avarage peak area
80000
60000
40000
20000
0
12h
24h
36h
**
*

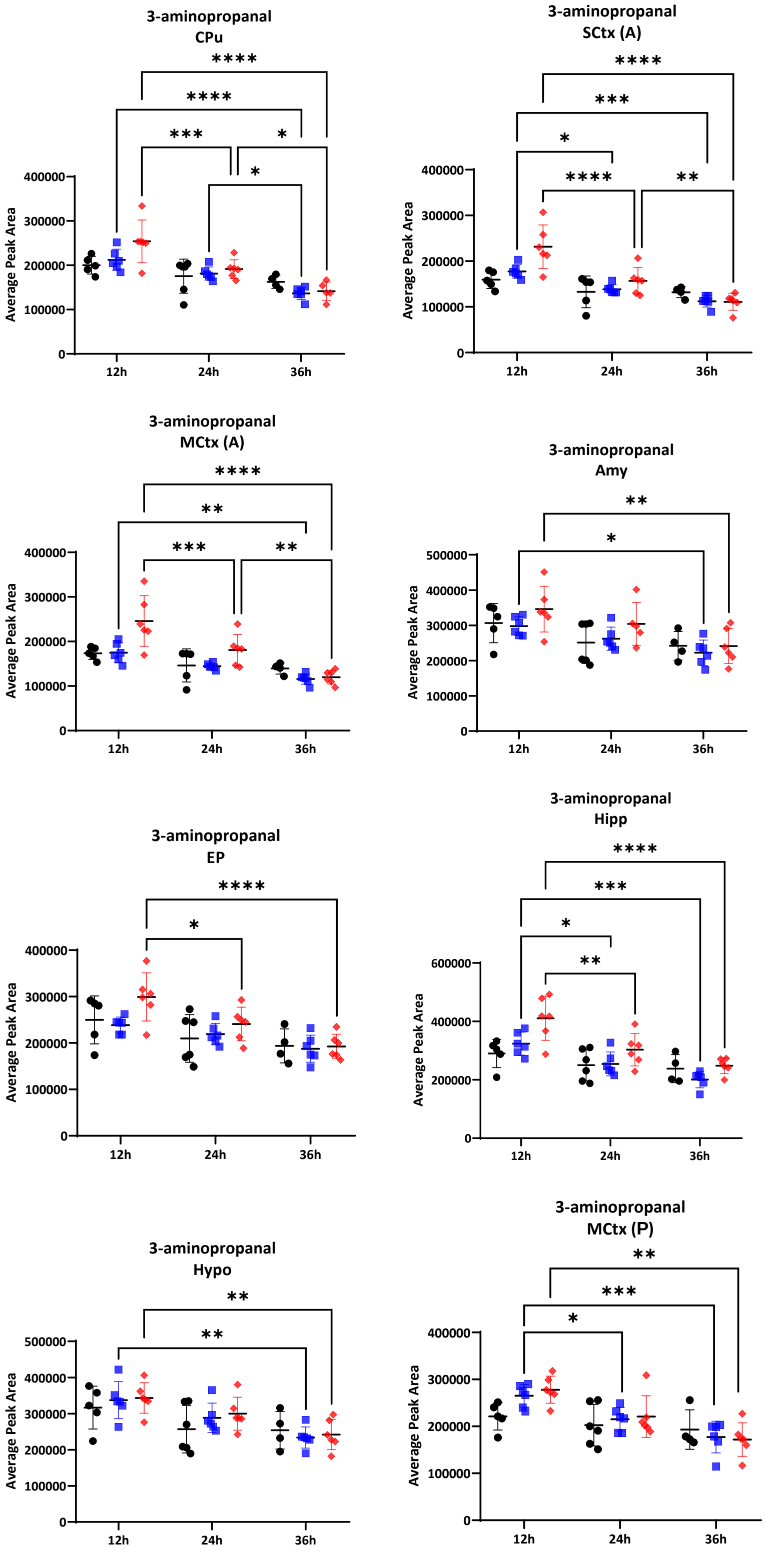
3-aminopropanal
CPu
3-aminopropanal
SCtx (A)
3-aminopropanal
MCtx (A)
3-aminopropanal
Amy
3-aminopropanal
EP
3-aminopropanal
Hipp
3-aminopropanal
Hypo
3-aminopropanal
MCtx (P)
Average Peak Area
12h
24h
36h
****
***
**
*

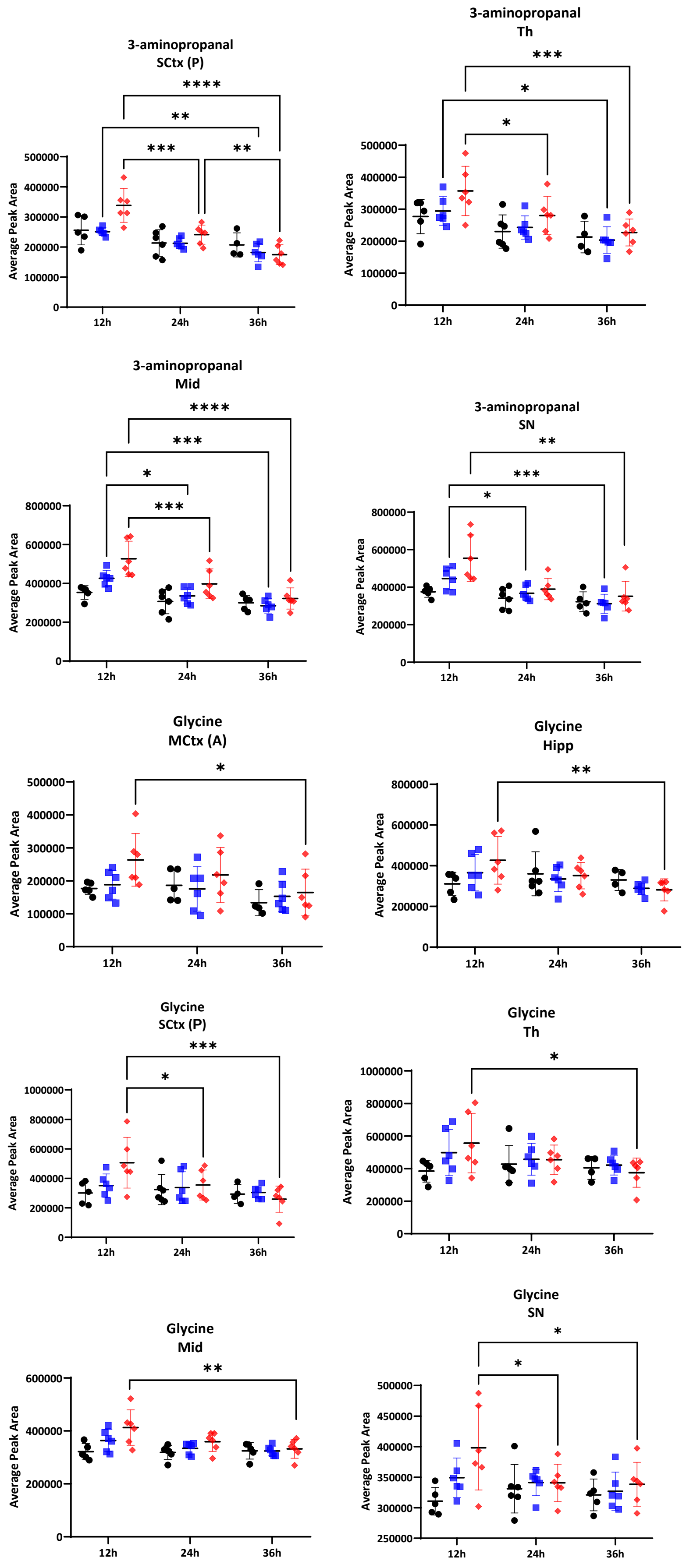
3-aminopropanal SCtx (P)
3-aminopropanal Th
3-aminopropanal Mid
3-aminopropanal SN
Glycine MCtx (A)
Glycine Hipp
Glycine SCtx (P)
Glycine Th
Glycine Mid
Glycine SN
Average Peak Area
12h
24h
36h

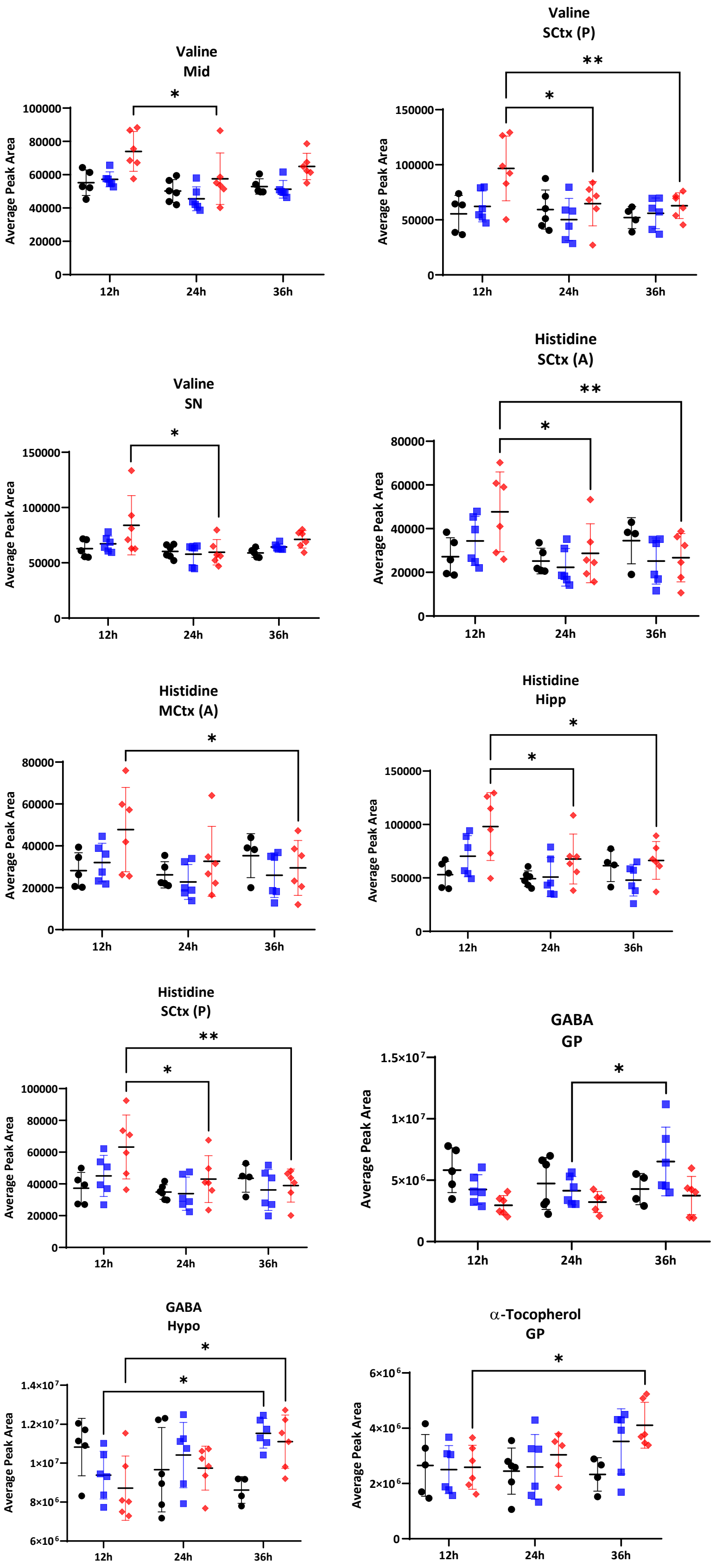
Valine
Mid
Valine
SCtx (P)
Valine
SN
Histidine
SCtx (A)
Histidine
MCtx (A)
Histidine
Hipp
Histidine
SCtx (P)
GABA
GP
GABA
Hypo
α-Tocopherol
GP
Average Peak Area
12h
24h
36h
*
**

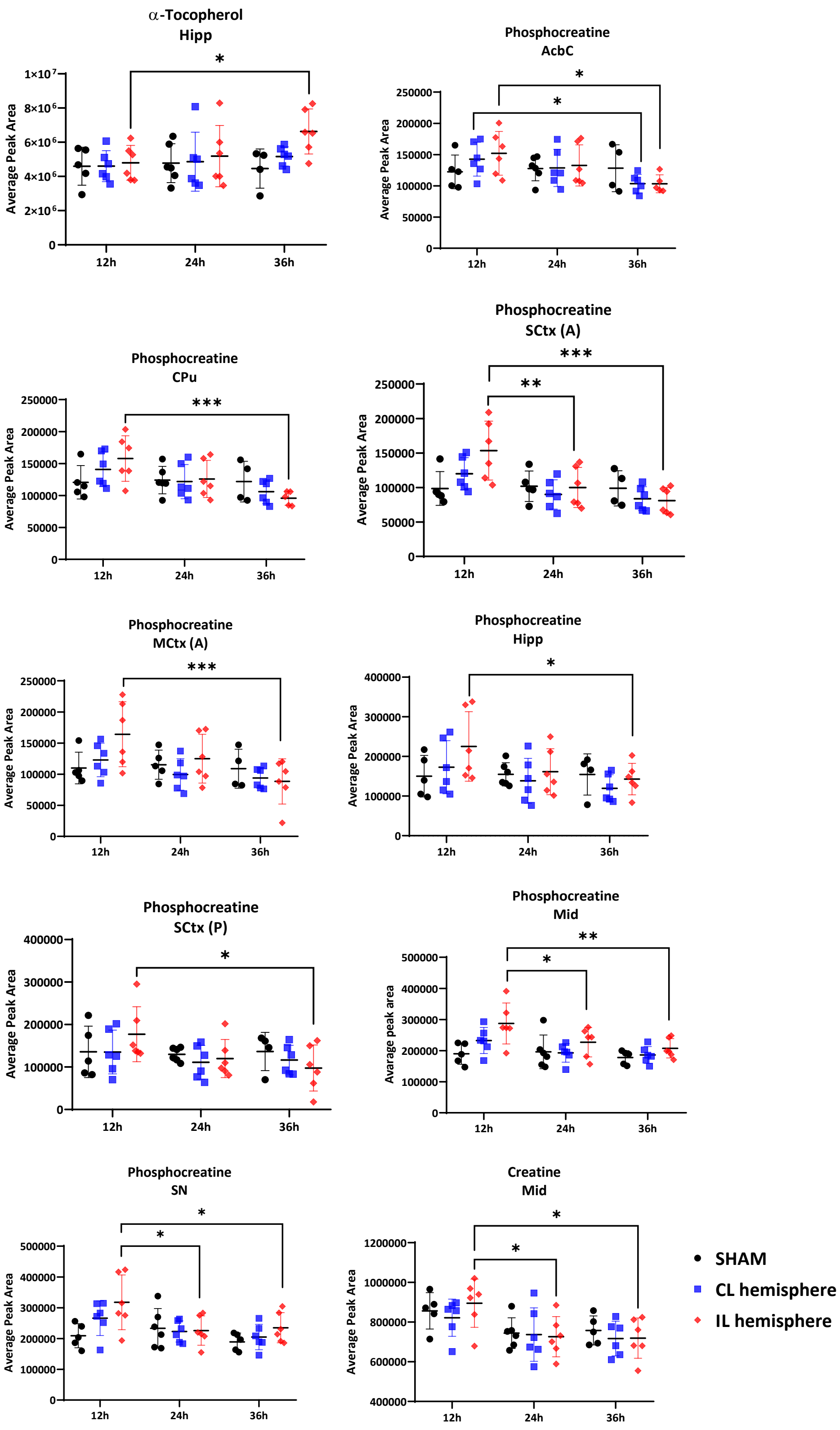

Figure S2. **Time courses of relative intensity levels of compounds detected in indicated brain regions.** Points indicate individual values (for sham brains in black, IL hemispheres in red; and CL hemispheres in blue); bars indicate means ± standard deviation (n=4-6 biological replicates; for details, see the Materials and Methods section). Data were normalized to internal standard, described in detail in Table S2. Note: nonparametric data, particularly pertaining to

3-aminopropanal in MCtx (P) and cadaverine in MCtx (A), were processed in injury-based status groups by Kruskal-Wallis tests or one-way ANOVA. *$p \leq 0.05$; **$p \leq 0.01$; ***$p \leq 0.001$; ****$p \leq 0.0001$. A, anterior level; P, posterior level; IL, ipsilateral; CL, contralateral; Hypo, hypothalamic region; GP, globus pallidus; Th, thalamic region; Amy, amygdala; MCtx, motor cortex; EP, entopeduncular nucleus; SCtx, somatosensory cortex; CPu, caudate putamen; Hipp, hippocampus; AcbC, accumbens nucleus core; SN, substantia nigra; Mid, midbrain; GABA, *γ*-aminobutyric acid, 5-HIAA, 5-hydroxyindoleacetic acid.

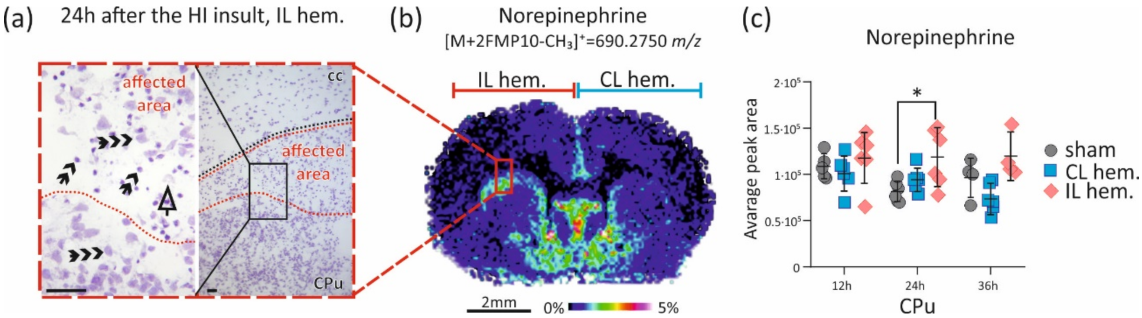

Figure S3. Intraregional increases in norepinephrine levels related to neurodegeneration in the neonatal rat brain after hypoxic-ischemic (HI) insult. (a) Nissl-stained section of dorsal caudate putamen (CPu), showing neuronal cell death at 24 hours post-insult; the scale bar represents 50 µm. (b) MALDI-MSI image of norepinephrine distribution in a striatal level brain section. Data were collected with a lateral step size of 100 µm. Data were normalized to dopamine-*d*4 internal standard. (c) Graph showing the relative intensity levels of norepinephrine in the entire CPu region of control (sham) and HI insult animals 12, 24, and 36 hours post insult. Results of two-way ANOVA with Tukey's multiple comparisons post hoc test (n=4–6 biological replicates, for details, see the Materials and Methods section); error bars indicate SD: *$p \leq 0.05$. Symbols: triple arrows, neuronal cells; triangular arrow, neuronal debris; double arrows, apoptotic cells. Abbreviations: cc, corpus callosum; CPu, caudate putamen; CL hem., contralateral hemisphere; IL hem., ipsilateral hemisphere.

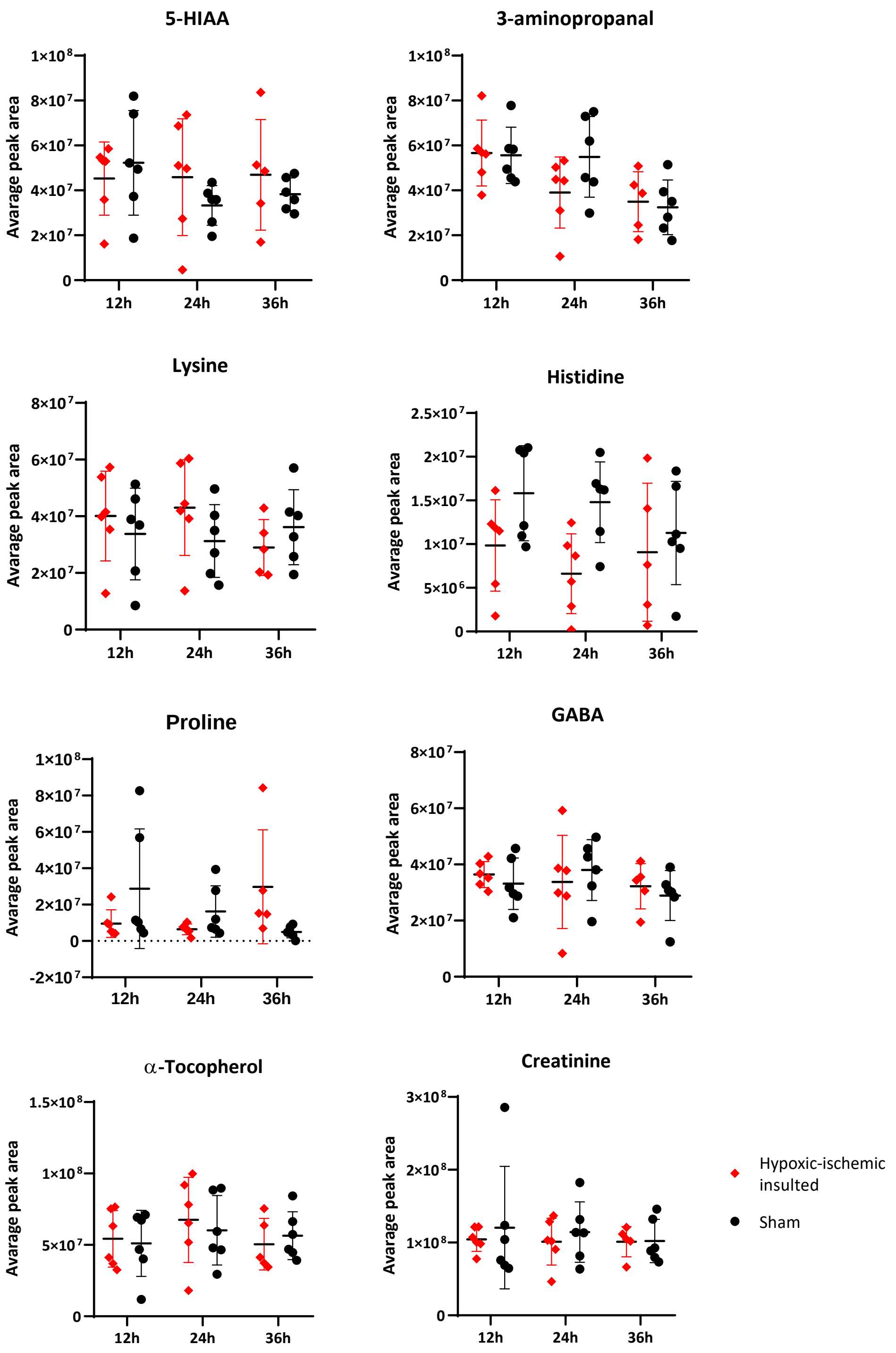

Figure S4. **Time courses of relative intensity levels of the compounds detected in cerebrospinal fluid**. Data were normalized to internal standard, described in detail in Table S2. Points indicate individual values (sham in black, HI-insulted in red); bars indicate means ± SD (*n*=4-6 biological replicates). 5-HIAA, 5-hydroxyindoleacetic acid; GABA, *γ*-aminobutyric acid.

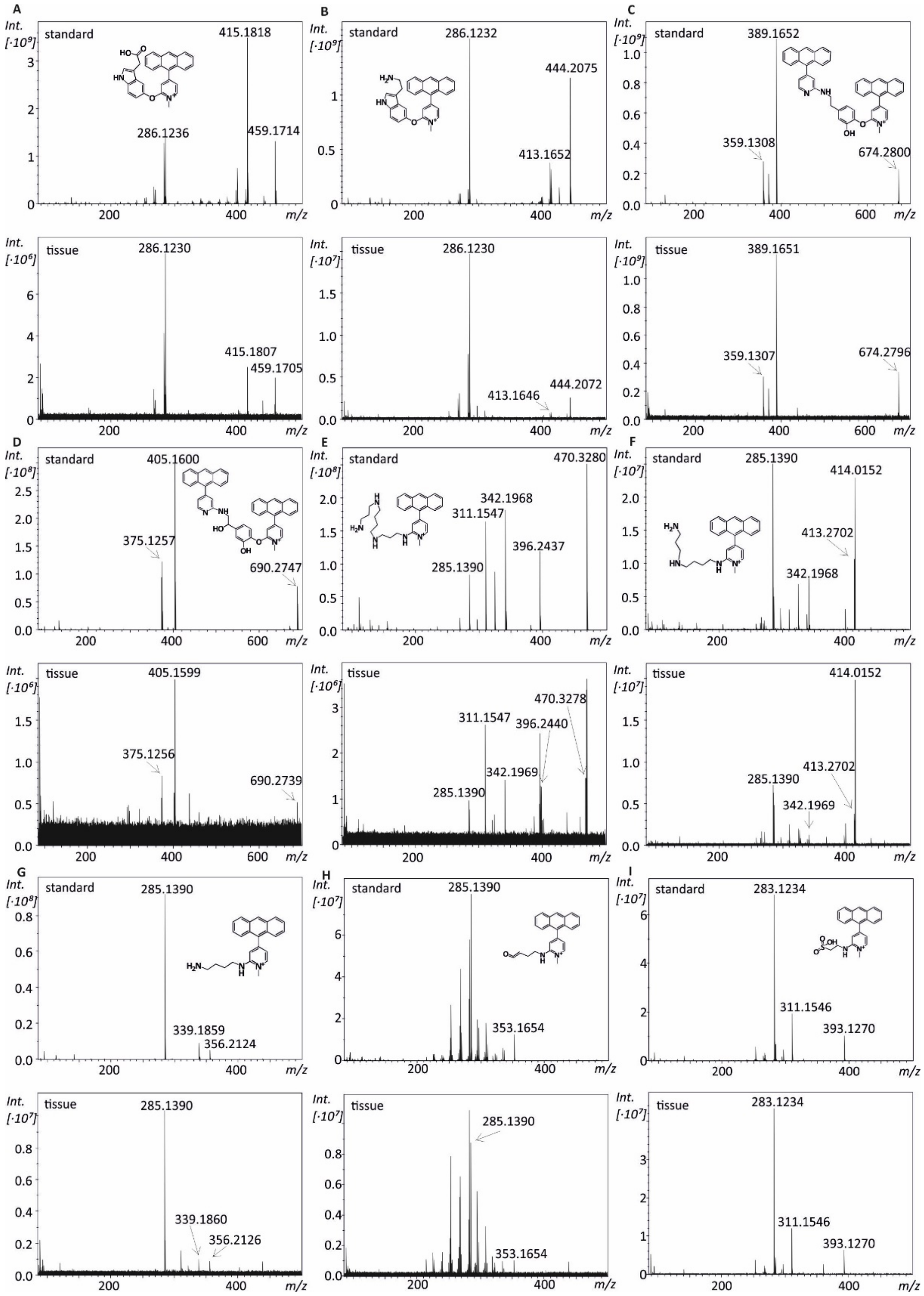
A
B
C
D
E
F
G
H
I
Int.
[·10^9]
[·10^6]
[·10^7]
[·10^8]
standard
tissue
m/z
415.1818
286.1236
459.1714
286.1230
415.1807
459.1705
286.1232
444.2075
413.1652
413.1646
444.2072
389.1652
359.1308
674.2800
389.1651
359.1307
674.2796
405.1600
375.1257
690.2747
405.1599
375.1256
690.2739
470.3280
342.1968
311.1547
396.2437
285.1390
470.3278
396.2440
342.1969
414.0152
413.2702
339.1859
356.2124
339.1860
356.2126
353.1654
283.1234
311.1546
393.1270

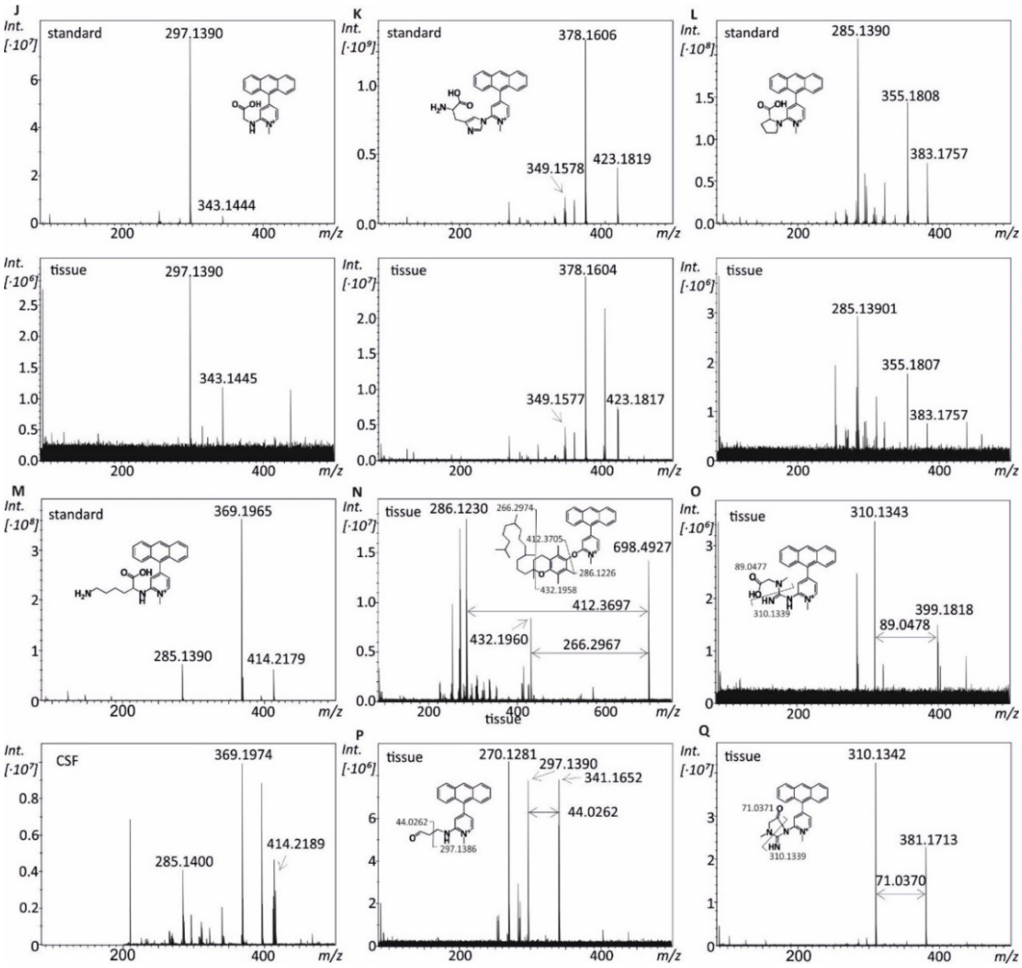

Figure S5. **Fragmentation spectra of the analyzed compounds.** Fragmentation pattern of commercial standards of 5-hydroxyindoleacetic acid (A, CV=35V), 5-hydroxytryptamine (B, CV=30V), dopamine (C, CV=30V), norepinephrine (D, CV=30V), spermine (E, CV=25V), spermidine (F, CV=25V), putrescine (G, CV=20V), γ-aminobutyric acid with in-source fragmentation loss of H_2O (H, CV=35V), taurine (I, CV=30V), glycine (J, CV=20V), histidine (K, CV=20V), proline (L, CV=30V) compared to on-tissue fragmentation patterns of in-brain detected compounds. In case of lysine (M, CV=20V), the fragmentation pattern of the commercial standard was compared with the compound detected in a CSF sample. 3-aminopropanal (P, CV=20V), α-tocopherol (N, CV=35V), creatine (O, CV=20V), and creatinine (P, CV=20V), for which no commercial standards were available, were characterized based on the characteristic neutral losses. 3-methoxytyramine, epinephrine, valine, and cadaverine were identified based on comparison of their accurate masses with accurate masses of the respective standards.

Table S1. **Phosphocreatine (PCr)/creatine (Cr) ratio.** The ratio PCr/Cr was normalized to the lowest obtained value (ratio of IL GP; *n*=4-6 biological replicates; for details, see the Materials and Methods section). CPu, caudate putamen; AcbC, accumbens nucleus core; SCtx (A), anterior somatosensory cortex; MCtx (A), anterior motor cortex; Amy, amygdala; EP, entopeduncular nucleus; GP, globus palidus; Hipp, hippocampus; Hypo, hypothalamic region; MCtc (P), posterior motor cortex; SCtx (P), posterior somatosensory cortex; Th, thalamic region; Mid, midbrain; SN, substantia nigra; CL, contralateral hemisphere; IL, ipsilateral hemisphere.

Timepoint and status	CPu	AcbC	SCtx (A)	MCtx (A)	Amy	EP	GP	Hipp	Hypo	MCtx (P)	SCtx (P)	Th	Mid	SN
12h														
SHAM	3.15	3.14	2.92	2.83	3.08	2.02	1.87	2.65	3.10	1.99	2.35	2.28	5.29	5.39
IL	4.20	3.88	4.23	4.41	2.78	1.98	1.36	3.29	2.54	2.20	2.67	2.45	7.66	8.98
CL	3.63	3.61	3.10	3.36	1.92	1.29	1.10	2.19	2.38	2.06	1.63	1.73	6.75	7.52
24h														
SHAM	3.64	3.57	3.23	3.03	2.22	1.51	1.20	2.14	2.16	1.79	1.89	1.80	6.29	6.43
IL	3.84	3.76	3.82	3.48	2.36	1.71	1.00	2.72	2.42	2.00	2.10	2.03	7.46	7.35
CL	3.44	3.72	2.93	2.91	2.04	1.38	1.00	2.10	2.20	1.78	1.59	1.65	6.24	6.96
36h														
SHAM	3.96	4.09	4.53	4.33	2.44	1.57	1.19	2.18	2.50	1.76	1.87	1.84	5.60	5.43
IL	2.52	2.39	2.78	2.43	2.39	1.70	1.38	2.45	2.29	1.78	2.15	2.16	6.89	6.82
CL	2.69	2.48	2.49	2.39	2.15	1.38	1.40	1.89	2.18	1.66	1.87	1.64	6.19	6.33

Table S2. **Normalizations used for all detected compounds.**

Normalization	*γ*-aminobutyric acid-*d6*	dopamine-*d4*	5-hydroxytryptamine-*d4*	Root Mean Square
Analyte	*γ*-aminobutyric acid	dopamine	5-hydroxyindoleacetic acid	histidine
	putrescine	3-methoxytyramine	5-hydroxytryptamine	histamine
	proline	epinephrine		
	taurine	norepinephrine		
	spermidine			
	glycine			
	valine			
	lysine			
	cadaverine			
	3-aminopropanal			
	α-tocopherole			
	creatine			
	creatinine			
	phosphocreatine			

Table S3. Reproducibility table. Coefficient of variance represents reproducibility. If a number is missing in a box, the compound was not measured or detected in a particular region/cerebrospinal fluid (CSF). Amy, amygdala; CPu, caudate putamen; EP, entopeduncular nucleus; GP, globus palidus; Hipp, hippocampus; Hypo, hypothalamic region; MCtx (P), posterior motor cortex; SCtx (P), posterior somatosensory cortex; Th, thalamic region.

Compound	Brain regions									CSF
	Amy	CPu	EP	GP	Hipp	Hypo	MCtx (P)	SCtx (P)	Th	
Dopamine +2[FMP-10]-CH_3			7.53							
3-methoxytyramine +FMP-10			23.43							
Norepinephrine +2[FMP-10]-CH_3			12.59							
Epinephrine +2[FMP-10]-CH_3			26.92							
5-hydroxyindoleacetic acid +FMP-10	11.90	14.45	17.25	11.87	8.68	13.97	9.26	11.13	14.76	21.22
5-hydroxytryptamine +FMP-10	5.22	13.29	17.86	20.05	10.45	9.63	10.69	4.84	15.12	
Spermine +FMP-10	18.31	13.65	14.30	15.19	6.59	17.54	1.53	13.58	11.58	
Spermidine +FMP-10	11.82	7.15	9.72	14.37	1.89	11.72	1.37	11.95	7.15	
Putrescine +FMP-10	7.88	3.89	4.30	10.18	0.91	12.32	4.96	8.09	3.06	
3-aminopropanal +FMP-10	3.43	5.70	3.55	9.91	5.15	9.18	4.57	2.34	2.75	12.60
Cadaverine +FMP-10	25.78	8.32	14.53	10.92	5.38	9.68	7.61	4.41	14.88	
Glycine +FMP-10	6.79	5.58	5.44	3.74	3.04	8.17	1.79	7.50	3.93	
Lysine +FMP-10	9.63	11.56	12.11	11.18	7.90	10.32	5.60	3.62	10.73	
Valine +FMP-10	8.48	1.57	6.75	12.79	1.37	6.38	2.38	2.82	3.32	16.53
Histidine +FMP-10	8.62	1.40	2.90	9.24	1.56	9.62	3.44	1.81	1.59	13.09
Proline +FMP-10	15.08	14.78	16.74	16.85	16.01	11.43	9.91	4.70	14.35	5.27
γ-aminobutyric acid -H_2O +FMP-10	9.19	10.14	15.21	17.28	10.81	10.26	9.57	4.89	8.10	8.30
Taurine +FMP-10	10.60	9.19	7.64	12.46	8.70	5.78	9.51	6.18	7.93	
α-Tocopherol +FMP-10	5.75	4.98	3.39	18.49	2.24	8.73	2.19	4.65	3.27	19.52
Creatine +FMP-10	10.66	23.93	24.43	13.57	22.76	17.81	19.18	19.79	21.70	
Creatinine +FMP-10	2.36	5.61	6.66	11.72	5.26	1.33	5.62	4.73	6.01	14.15
Phosphocreatine-H_2O+FMP-10	2.90	9.03	5.17	11.05	8.46	7.56	6.49	4.42	8.06	
